# Repeat-Rich Architecture and Gene Duplication Characterize the Expanded Genome of the Chestnut Gall Wasp

**DOI:** 10.3390/insects17101047

**Published:** 2026-10-12

**Authors:** Ye-Song Ren, An-Kang Lv, Yang Zeng, Dao-Hong Zhu

**Affiliations:** Laboratory of Insect Behavior and Evolutionary Ecology, College of Life Sciences, Central South University of Forestry and Technology, Changsha 410004, China; 20230100052@csuft.edu.cn (Y.-S.R.); yl240019@126.com (A.-K.L.)

**Keywords:** *Dryocosmus kuriphilus*, genome expansion, transposable elements, gene duplication, parthenogenesis, environmental adaptation

## Abstract

*Dryocosmus kuriphilus*, the chestnut gall wasp, is a globally invasive pest that reproduces by thelytokous parthenogenesis. Despite the absence of conventional sexual recombination, the species has spread widely and persists under diverse environmental conditions, but the genomic features associated with this capacity remain poorly understood. Here, a chromosome-level assembly of *D. kuriphilus* was compared with seven representative insect genomes, with emphasis on repetitive sequences, orthogroups, and gene-duplication patterns. The 2.28-Gb *D. kuriphilus* genome contained 79.7% repetitive sequences, including 62.3% transposable elements (TEs), most of which occurred in intergenic regions. OrthoFinder assigned 13,271 *D. kuriphilus* genes to multi-copy orthogroups, representing 61.5% of the protein-coding gene set. Independent MCScanX analysis classified genes into mutually exclusive dispersed, proximal, tandem, and segmental duplication modes, with segmental and dispersed representing the two largest duplicated-gene classes. *D. kuriphilus* contained substantially more segmental duplicates than the other species examined. Synonymous-substitution distributions revealed low- and high-Ks components representing different levels of synonymous divergence among duplicated genes, and genes in these components showed distinct functional profiles. Numerous local duplications were found near TEs and included genes involved in host recognition, nutrient utilisation, xenobiotic metabolism, and energy production. Compared with the closely related gall wasp *Belonocnema kinseyi*, *D. kuriphilus* has a broader reported distribution and host range and contains higher copy numbers in several gene families associated with chemosensation, detoxification, metabolism, and stress responses. Together, these findings show that the expanded *D. kuriphilus* genome is characterized by extensive repeat accumulation and increased gene copy number, whereas duplication at multiple scales increased and reshaped the functional gene repertoire. These genomic features identify candidate mechanisms that may contribute to genomic and ecological plasticity, although their adaptive significance requires expression, evolutionary, and functional validation.

## Simple Summary

The chestnut gall wasp is a serious invasive pest that damages chestnut trees and reduces nut production. Because it can reproduce without mating, even a single female may establish a new population, yet the genetic features that may support its spread and survival in different environments remain unclear. We compared its genome, the insect’s full set of genetic information, with those of seven other insects to investigate why it is unusually large and how its genes have changed. Nearly four-fifths of the genome consisted of repeated sequences, and more than three-fifths of its protein-producing genes were present in multiple copies. Many of these genes were linked to finding host plants, processing food, removing harmful chemicals, producing energy, and coping with environmental stress. The unusually large genome was associated with a high abundance of repeated sequences, while extra gene copies expanded the range of functions represented in the genome. This study provides a foundation for investigating how this pest spreads and adapts and may help guide future monitoring and control efforts in chestnut-growing regions.

## 1. Introduction

*Dryocosmus kuriphilus* Yasumatsu, the Oriental chestnut gall wasp, is a globally invasive pest of *Castanea* species and is native to China. It attacks most cultivated chestnuts, including Chinese, Japanese, European, and American chestnuts [1,2,3,4]. The species spread from China to Japan and the Republic of Korea before reaching North America and Europe. By February 2026, the EPPO Global Database recorded the pest in at least 28 countries and territories across Asia, Europe, and North America [5].

Long-distance dispersal occurs mainly through the movement of infested nursery stock, scions, and other propagative material because eggs and early-instar larvae remain concealed within buds [4,6,7,8,9]. Thelytokous parthenogenesis further promotes establishment because a single female can found a population and produce up to 300 eggs [10,11]. Natural spread at invasion fronts is approximately 7 km per year, whereas human-mediated jump dispersal can average up to 76 km [6,12].

Gall formation reduces shoot elongation, leaf expansion, female flower development, and nut production. Reported annual yield losses range from 15–30% in China and 50–75% in the United States to as much as 80% in severely infested Italian orchards [13,14,15]. Consequently, *D. kuriphilus* is recognised as one of the most destructive pests of chestnut production and is listed by the European and Mediterranean Plant Protection Organization as a quarantine pest [3,16].

Its concealed development, parthenogenetic reproduction, and dispersal efficiency enable *D. kuriphilus* to establish rapidly in invaded regions. Studies of its life cycle, population dynamics, dispersal history, and interactions with chestnut-associated organisms document persistence across diverse climates and management contexts [17,18,19,20,21]. Identifying genomic features that may contribute to this ecological plasticity is therefore important for understanding the invasion success of the species.

Parthenogenesis is a defining reproductive feature of *D. kuriphilus*. In Hymenoptera, transitions to thelytokous parthenogenesis have occurred repeatedly, and thelytoky can be obligate, facultative, or sporadic and can arise through either apomictic or automictic mechanisms [22]. These mechanisms differ in their effects on meiotic recombination and the maintenance of heterozygosity, and the precise cytological basis of thelytoky remains unresolved for many hymenopteran species. Compared with conventional sexual reproduction, thelytokous reproduction generally reduces opportunities for allelic reshuffling and the formation of novel genotypic combinations, which may constrain the supply of genetic variation available for adaptation, particularly under rapidly changing conditions [23,24]. Nevertheless, many parthenogenetic or recombination-limited species retain substantial ecological plasticity and invasion capacity, indicating that alternative sources of genomic variation may contribute to their evolutionary potential [25,26,27].

Gene duplication and transposable elements (TEs) are two major sources of genomic variation [28,29]. Duplication can alter gene dosage and enable the retention, partitioning, or acquisition of gene functions [30,31,32,33]. TE activity can modify gene structure and expression through insertion and can also promote non-allelic homologous recombination and structural variants, including deletions, inversions, translocations, and duplications [29,34,35,36]. In insects, expansions of gene families associated with detoxification, chemosensation, immunity, and stress responses frequently accompany duplication or TE-mediated genomic remodelling [37,38,39].

A chromosome-level genome assembly is available for *D. kuriphilus*, providing a foundation for examining the relationship between parthenogenesis, repeat accumulation, gene duplication, and genomic plasticity [40]. Here, chromosome-level genomes from eight insect species were compared to quantify repeat content and distribution, reconstruct orthogroups and gene-family changes, classify duplication modes, and assess the functional composition of duplicated-gene groups. The analyses were designed to determine how repeat expansion and duplication at different genomic scales have shaped the *D. kuriphilus* genome and its repertoire of genes associated with host use and environmental responses.

## 2. Methods

**Genome data sources.** Chromosome-level genome assemblies were downloaded from InsectBase 2.0 (http://v2.insect-genome.com/, accessed on 16 August 2026) for comparison with *D. kuriphilus*. The dataset comprised the dipteran *Drosophila melanogaster*, the coleopteran *Tribolium castaneum*, the lepidopteran *Bombyx mori*, and the hymenopterans *Nasonia vitripennis*, *Ooceraea biroi*, *Apis mellifera*, and *Belonocnema kinseyi* (Table 1).

**Genomic data processing.** The downloaded datasets included chromosome-level genome sequences in FASTA format, gene annotations with chromosomal coordinates in GFF format, predicted protein sequences, and coding sequences. For loci with alternative transcripts, only the longest isoform was retained to reduce redundancy and improve comparability among species. Coding sequences whose lengths were not divisible by three or that lacked complete start or stop codons were excluded as potentially incomplete or problematic gene models. These filtering criteria were used for sequence-quality control and were not interpreted as evidence of pseudogene status. The corresponding protein and coding sequences were then extracted to generate a non-redundant, standardised gene set for each species. For transparency, the numbers of gene models excluded by this filtering procedure were recorded for each species and are provided in Supplementary Table S7.

**Orthogroup construction and copy-number analysis.** Orthologous and paralogous genes within and among species were identified using OrthoFinder v2.5.2 [41]. An in-house pipeline was used to count the number of genes contributed by each species to each orthogroup. Genes were summarized according to their within-species copy number using five descriptive classes: single copy, 2–10 copies, 11–20 copies, 21–100 copies, and >100 copies. Orthogroups containing more than 100 copies in a given species are referred to here as high-copy orthogroups. The >100-copy threshold was used solely as an operational category for describing the upper tail of the copy-number distribution and was not interpreted as evidence of evolutionary gene-family expansion. No inferential statistical test was applied to these descriptive copy-number classes. Evolutionary gene-family expansion and contraction were evaluated separately using the phylogenetic CAFE analysis described below.

**Identification and classification of duplicated genes using MCScanX.** Gene-duplication modes were independently classified for each species using the duplicate_gene_classifier program implemented in MCScanX [42]. For each genome, the standardized protein-coding gene set and corresponding genomic coordinates were used together with within-genome protein similarity information generated by self-BLASTP. MCScanX collinearity analysis was performed before running duplicate_gene_classifier.

The final *.gene_type output generated by duplicate_gene_classifier was used as the definitive gene-level duplication classification in all subsequent duplication-mode analyses. Each gene was assigned to a single, mutually exclusive category according to the hierarchical MCScanX classification procedure: segmental/collinear duplication had priority over tandem duplication, followed by proximal and dispersed duplication. Genes without detectable duplicated homologues were classified as singletons. The final gene-type codes were therefore interpreted as 0, singleton; 1, dispersed duplicate; 2, proximal duplicate; 3, tandem duplicate; and 4, segmental duplicate.

The total number of MCScanX-classified duplicated genes was calculated as the sum of dispersed, proximal, tandem, and segmental genes, excluding singleton genes. Percentages of individual duplication modes were calculated relative to this MCScanX duplicated-gene total. These MCScanX classifications were kept separate from the OrthoFinder-based orthogroup copy-number analysis described above.

**Phylogenetic tree reconstruction.** Single-copy orthologues shared among the 8 species were identified from the OrthoFinder output. Amino-acid sequences in each orthogroup were aligned independently with MUSCLE v3.8.31 [43]. A custom C++ script converted the amino-acid alignments to codon-based nucleotide alignments by reference to the original coding sequences. The codon alignments were concatenated into a combined nucleotide matrix for the eight species.

A maximum-likelihood phylogeny was reconstructed from the concatenated matrix with PhyML v3.0 [44], and divergence times were estimated with MCMCTree in PAML v4.9 [45]. Three constraints from TimeTree were used for calibration: 151–344 million years ago (Ma) for *Bombyx mori* and *Drosophila melanogaster*, 100–163 Ma for *Apis mellifera* and *Ooceraea biroi*, and 310–389 Ma for *Tribolium castaneum* and *Bombyx mori*.

**Gene-family expansion and contraction.** Changes in gene-family size were analysed from the OrthoFinder copy-number matrix and the ultrametric species tree using CAFE v4.2.1, which models gene-family gain and loss under a stochastic birth–death process [46]. Prior to CAFE analysis, orthogroups containing >100 copies in any species were excluded to prevent exceptionally large families from disproportionately affecting estimation of the global gain–loss rate parameter (λ). The remaining families were used to estimate λ and reconstruct ancestral gene-family sizes across the phylogeny. For each branch, a family was classified as expanded when its inferred copy number at the descendant node was greater than that at the immediate ancestral node, and as contracted when the inferred copy number was lower. These branchwise increases and decreases, rather than families selected using the CAFE family-wide *p*-value, were used to summarize gene-family expansion and contraction. Accordingly, no multiple-testing correction was applied to the CAFE family-wide *p*-values because these *p*-values were not used to define the reported expanded or contracted families. The 670 families reported for *D. kuriphilus* therefore represent families inferred to have increased in copy number along the terminal branch leading to *D. kuriphilus*, rather than statistically significant or *D. kuriphilus*-specific gene families. These terminal-branch expanded families were subsequently subjected to KEGG enrichment analysis.

**Functional annotation and enrichment.** Protein sequences were queried with BLASTP v2.3.0+ against the Gene Ontology (GO) and Kyoto Encyclopedia of Genes and Genomes (KEGG) databases at an E-value threshold of 1 × 10^−5^. GO and KEGG enrichment analyses were conducted with OmicShare and WEGO v2.0, respectively, and results were visualised in R with ggplot2. To compare the relative representation of functional categories among duplicate groups, a functional representation index (FRI) was calculated for each KEGG category. For duplication group i and functional category j, FRI was defined as FRI_ij_ = (n_ij_/N_i_)/(n_j_/N), where n_ij_ is the number of genes assigned to category j in group i, N_i_ is the total number of functionally annotated genes in group i, n_j_ is the number of genes assigned to category j in the genomic reference background, and N is the total number of annotated genes in that background. The reference background comprised all KEGG-annotated protein-coding genes in the *D. kuriphilus* genome. FRI values were log2-transformed, with positive and negative values indicating relative over-representation and under-representation, respectively. FRI values were calculated separately for the low-Ks (Ks = 0–0.2) and high-Ks (Ks = 2.2–2.5) components and for dispersed, proximal, tandem, and segmental duplicates.

For the FRI analysis, statistical over-representation of each KEGG category relative to the genomic reference background was evaluated using a one-sided hypergeometric test. The resulting *p*-values were adjusted for multiple testing using the Benjamini–Hochberg procedure, and categories with an adjusted *p* < 0.05 were considered significantly over-represented relative to the genomic background. These tests evaluated each duplicate group relative to the genomic background and did not directly compare the low-Ks and high-Ks components.

To describe differences in functional representation between the two Ks components, the difference in log2(FRI) was calculated as Δlog2(FRI) = log2(FRI_low-Ks) − log2(FRI_high-Ks). Functional categories showing greater log2(FRI) values in the low-Ks component were described as low-Ks-biased, whereas those showing greater values in the high-Ks component were described as high-Ks-biased. Categories showing relatively similar values between the two components were described as weakly differentiated. These classifications were based on descriptive differences in relative representation and did not imply statistically significant differences between the two components. No direct statistical test comparing functional representation between the low-Ks and high-Ks components was performed.

**Functional enrichment of TE-associated tandem duplicates.** To investigate the functional characteristics of duplicated genes associated with TE-containing co-duplicated regions, KEGG and GO enrichment analyses were performed for the TE-associated tandem-duplicate gene set. All annotated protein-coding genes of *D. kuriphilus* were used as the reference background. Over-representation of functional categories was tested using the hypergeometric test, followed by Benjamini–Hochberg correction for multiple testing. Functional categories with an adjusted *p* < 0.05 were considered significantly enriched.

**Repeat annotation.** Repetitive sequences were annotated using complementary approaches for interspersed and tandem repeats. Tandem repeats were identified with Tandem Repeats Finder v4.07b [47]. Interspersed transposable elements (TEs) were annotated using RepeatModeler v1.0.4 [48] and RepeatMasker v4.0.6 [49]. Species-specific repeat libraries were generated de novo with RepeatModeler and classified against TEclass/Repbase, after which RepeatMasker was used to identify known and novel TE sequences. In this study, “TEs” refer to interspersed repeat annotations generated by the RepeatModeler/RepeatMasker pipeline, whereas “tandem repeats” refer to tandem arrays identified independently by Tandem Repeats Finder; both categories are included under the broader term “repetitive sequences.” To avoid double counting, overlapping intervals between TE and tandem-repeat annotations were merged, and total repeat length was calculated from the union of non-overlapping repeat-covered genomic intervals. Repeat proportions were calculated as the non-redundant repeat-covered length divided by total assembly length.

**Identification of TE-associated gene co-duplication.** Gene-duplication events were first identified from the OrthoFinder analysis. To characterize the spatial relationships between duplicated genes and nearby transposable elements, the genomic positions of duplicated genes were integrated with the TE annotations generated by RepeatMasker. For each duplicated gene pair associated with homologous TE copies, the corresponding genomic regions were divided into three components: the TE sequence, the intervening sequence between the TE and the gene, and the duplicated gene sequence. Corresponding components between duplicated loci were aligned at the nucleotide level, and sequence identity was calculated.

TE-associated duplicated regions were classified into two structural types. Type I regions were defined when the TE sequence, the intervening sequence, and the duplicated gene all showed >95% nucleotide identity between the duplicated loci, indicating co-duplication of a continuous TE–intervening-region–gene segment. Type II regions were defined when the TE and duplicated gene showed >95% nucleotide identity, whereas the intervening sequences showed substantially lower similarity.

The duplicated genes identified in these TE-associated structures were subsequently classified according to duplication mode using MCScanX, including tandem, proximal, dispersed, and segmental duplication. The relative contribution of each duplication mode was then summarized to describe which duplication classes were most frequently represented among TE-containing co-duplicated segments.

**Synonymous and nonsynonymous substitution rates.** Homologous gene pairs within the *D. kuriphilus* genome were obtained from the LAST all-versus-all protein-similarity results used as input for the MCScanX analysis. These homologous relationships were subsequently associated with the dispersed, proximal, tandem, and segmental duplication categories defined by MCScanX. Single-copy orthologous gene pairs between *D. kuriphilus* and each of the other seven species were identified using OrthoFinder. Synonymous substitution rates (Ks) were calculated using KaKs_Calculator v2.0 with default parameters [50].

Ks values were interpreted as indicators of relative synonymous divergence rather than as direct estimates of duplication age because they can be affected by substitution saturation, lineage-specific evolutionary rates, and variation among gene pairs. Kernel-density distributions were used to visualize the Ks distributions. The local density maxima corresponding to the lower- and higher-Ks concentrations were identified by visual inspection, and symmetric intervals around the respective density maxima were then selected to define representative subsets for downstream functional comparison. The resulting intervals were Ks = 0–0.2 for the low-Ks component and Ks = 2.2–2.5 for the high-Ks component. Thus, these boundaries were selected as equal-distance windows around the respective local density maxima rather than being estimated by a formal mixture model or clustering procedure. The two groups are therefore treated as operational Ks components for exploratory comparison and not as discrete duplication events or defined evolutionary periods.

Because homologous pairs were derived from an all-versus-all similarity search, large multi-copy gene families could contribute more pairwise comparisons than small families. No family-size weighting or subsampling was applied in the present analysis. We therefore interpret the Ks distributions and the subsequent functional comparisons descriptively and consider the comparisons between the low- and high-Ks components exploratory.

**Classification of gene-duplication types.** Gene duplications were identified and classified with the duplicate_gene_classifier script in MCScanX using default parameters [42]. Genes were assigned to five categories: singleton genes with no detectable duplicates; dispersed duplicates in non-collinear regions; proximal duplicates on the same chromosome and separated by no more than 10 genes; tandem duplicates on the same chromosome and separated by no more than three genes; and segmental duplicates.

**Identification of candidate gene families associated with host use and environmental responses.** Candidate gene families associated with chemosensation, detoxification, host use, energy metabolism, and environmental stress responses were compared between *D. kuriphilus* and *B. kinseyi*. Representative hymenopteran proteins with clear annotations were downloaded from the NCBI Protein database by combining the taxon term ‘Hymenoptera’ with each gene-family name. These sequences were queried against the standardised protein sets of the two gall wasps with BLASTP. Candidate homologues were retained at an E-value threshold of 1 × 10^−5^ and manually checked for sequence similarity and annotation consistency. Only the longest isoform per locus was retained, and redundant sequences, apparent partial models, and candidates with insufficient similarity were excluded. The analysed families comprised odorant receptors (ORs), ionotropic receptors (IRs), odorant-binding proteins (OBPs), cytochrome P450 monooxygenases (P450s), UDP-glycosyltransferases (UGTs), carboxyl/cholinesterases (CCEs), sulfotransferases (SULTs), major facilitator superfamily (MFS) transporters, aldehyde oxidases (AOXs), succinate dehydrogenases (SDHs), malate dehydrogenases (MDHs), adenylate kinases (AKs), pyruvate kinases (PKs), fatty acid synthases (FASs), heat-shock protein 70s (Hsp70s), superoxide dismutases (SODs), glutathione peroxidases (GPXs), transient receptor potential channels (TRPs), and aquaporins (AQPs). Non-redundant genes assigned to each family were counted separately for the two species. Gene-family copy numbers were used as descriptive comparative genomic features. Differences in copy number were not considered direct evidence of ecological adaptation, but were used to identify candidate families for subsequent evolutionary and functional interpretation.

## 3. Results

### 3.1. Large Cynipid Genomes Are Associated with Extensive Repeat Accumulation and Intergenic Expansion

To place the large *D. kuriphilus* genome in a broader phylogenetic context, published genome-size estimates were compared across hymenopteran families. Most available non-cynipid hymenopteran genomes were relatively small, with mean sizes of approximately 297 Mb in Apidae, 292 Mb in Formicidae, 381 Mb in Pteromalidae, and 266 Mb in Braconidae/Ichneumonidae (Supplementary Table S1). However, relatively large genomes also occur in Figitidae. The genome sizes of *Ganaspis brasiliensis* and *G. xanthopoda* are approximately 831 and 972 Mb, respectively, indicating that genome enlargement is not restricted to Cynipidae. Nevertheless, available genomes from the tribe Cynipini remain particularly large, averaging approximately 1720 Mb (Supplementary Figure S1). This broader pattern, together with the repeat-rich 1.12-Gb genome of *Neuroterus valhalla* and other large Cynipini genomes [51], indicates substantial genome-size variation across Cynipoidea, with pronounced genome expansion in several Cynipini lineages.

**Figure 1:**
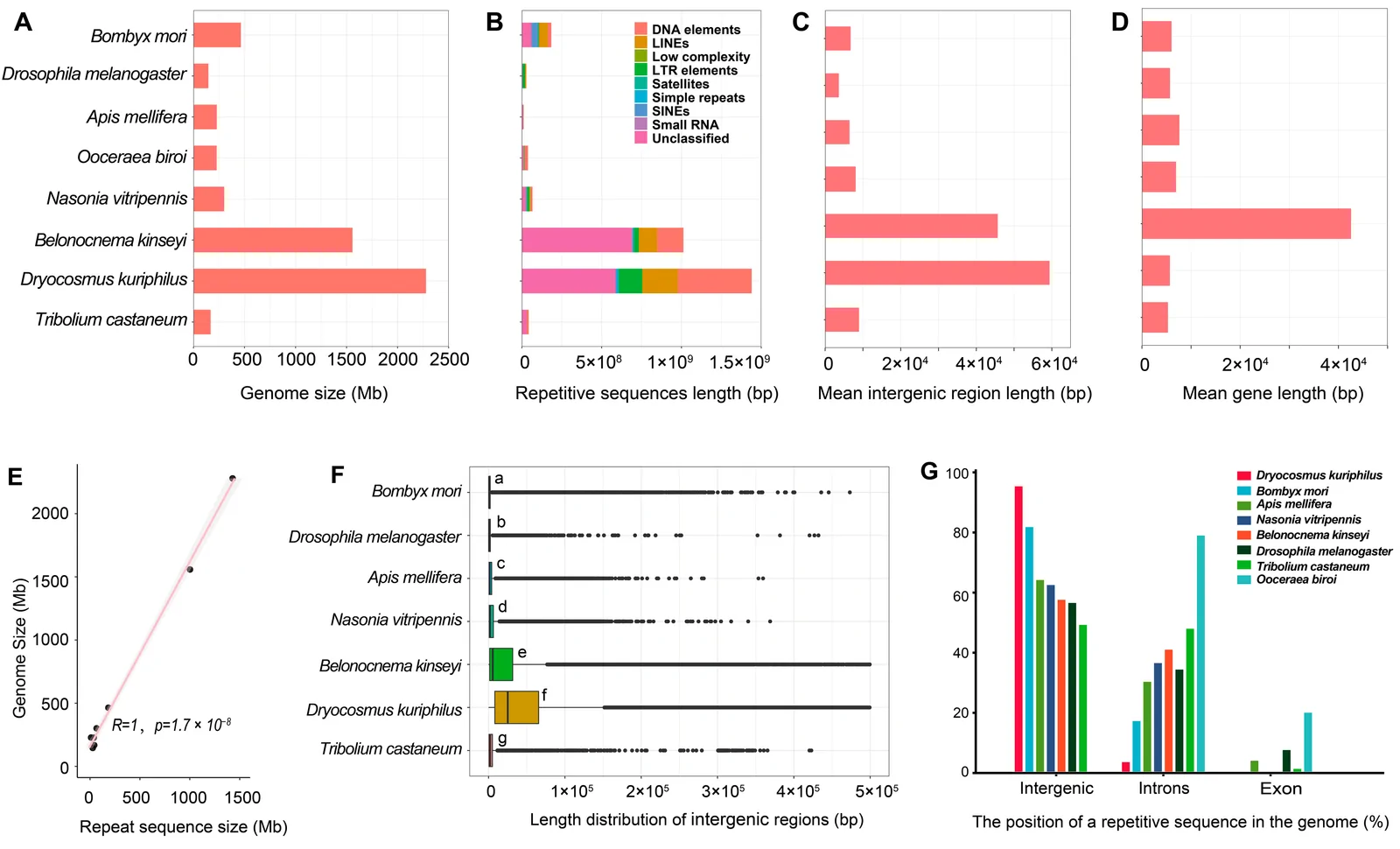
Genome size is associated with repeat content and intergenic elongation in cynipid gall wasps. (**A**) Genome sizes of the eight species. (**B**) Total length and composition of repetitive sequences, including DNA elements, LINEs, LTR retrotransposons, low-complexity regions, Satellites, Simple repeats, SINEs, Small RNAs, and unclassified repeats. (**C**) Mean intergenic-region length. (**D**) Mean gene length. (**E**) Descriptive relationship between genome size and total repeat length across the eight species. The pink line represents the fitted linear regression line. Because total repeat length is a component of genome size, this relationship is presented for descriptive visualization rather than as an independent statistical test of genome expansion. (**F**) Distribution of intergenic-region lengths across the seven species shown. The lowercase letters (a–g) correspond to the species listed from top to bottom and are used solely for species identification, not to indicate statistical significance. (**G**) Genomic distribution and proportional composition of repetitive sequences in the eight species.

Comparison of the eight focal genomes confirmed that the cynipid gall wasps *D. kuriphilus* and *B. kinseyi* had substantially larger genomes than the six non-cynipid insects (Table 2; Figure 1A). The *D. kuriphilus* genome was approximately 5–10 times larger than those of the six non-cynipid species and nearly 700 Mb larger than that of *B. kinseyi*. Thus, *D. kuriphilus* shows additional lineage-specific expansion against the broader background of large cynipid genomes.

The *D. kuriphilus* genome contained 1815.9 Mb of repetitive sequence, including TEs and tandem repeats, which together represented 79.7% of the assembly (Table 3). TEs and tandem repeats accounted for 62.3% and 17.4% of the genome, respectively. The B. kinseyi genome contained 1002.3 Mb of repetitive sequence, whereas the total TE content of each non-cynipid species did not exceed 66 Mb (Table 4; Figure 1B). Mean gene length in *D. kuriphilus* was 5676 bp, comparable to that of *D. melanogaster*, *T. castaneum*, *B. mori*, and *N. vitripennis* and shorter than that of *A. mellifera* and *O. biroi* (Figure 1D). By contrast, mean gene length in *B. kinseyi* was 42,564 bp, possibly reflecting repeat accumulation within introns. A descriptive comparison of genome size and total repeat length showed that species with larger genomes also contained greater absolute amounts of repetitive sequence (Figure 1E). Because repeat length is itself a component of total genome size, this relationship was interpreted descriptively rather than as an independent statistical test of genome expansion. Together with the markedly elongated intergenic regions of the two cynipid genomes, these observations indicate an association between large genome size and repeat-rich non-coding sequence, but do not establish repeat proliferation as the evolutionary cause of genome expansion. Because repeat length is a component of genome size, this correlation is expected and should be interpreted as an association rather than an independent test of causality. The data therefore show that the two cynipid genomes are repeat-rich and have elongated intergenic regions, but they do not establish the temporal direction or rate of repeat-driven genome expansion.

DNA transposons and unclassified TEs were the dominant repeat classes in *D. kuriphilus* and accounted for 20.3% and 25.8% of the genome, respectively. LINEs and LTR retrotransposons represented 9.8% and 6.5%, whereas SINEs accounted for only 0.4% (Figure 1B). The total lengths of DNA transposons, LINEs, and LTRs in *D. kuriphilus* were each more than twice those in *B. kinseyi*. *D. kuriphilus* therefore showed a greater expansion of classified TEs, whereas unclassified TEs contributed a larger proportion of the *B. kinseyi* repeat complement. Although the other six species differed in their dominant TE classes, their total TE lengths were substantially smaller than those of the two cynipid species (Supplementary Figure S2).

*D. kuriphilus* and *B. kinseyi* also had significantly longer intergenic regions than the six non-cynipid species (Kruskal–Wallis test followed by Dunn’s test, false-discovery-rate-adjusted *p* < 0.05; Figure 1C,F). Their mean intergenic lengths were at least 6.6-fold and 5.1-fold greater, respectively. Expansion of intergenic sequence is therefore a prominent structural feature of both enlarged cynipid genomes.

Nearly 95% of TEs in *D. kuriphilus* occurred in intergenic regions, where DNA transposons were the dominant class (Figure 1G; Supplementary Figure S3). TEs were similarly enriched in the intergenic regions of *B. kinseyi*. In species with shorter intergenic regions, approximately 50–60% of TEs occurred in intergenic regions and about 40% in introns (Figure 1G). The concentration of TEs in intergenic regions therefore coincided with extensive intergenic expansion in the two cynipid genomes.

### 3.2. Gene Duplication Remodels the Gene Repertoire of the Chestnut Gall Wasp

**Figure 2:**
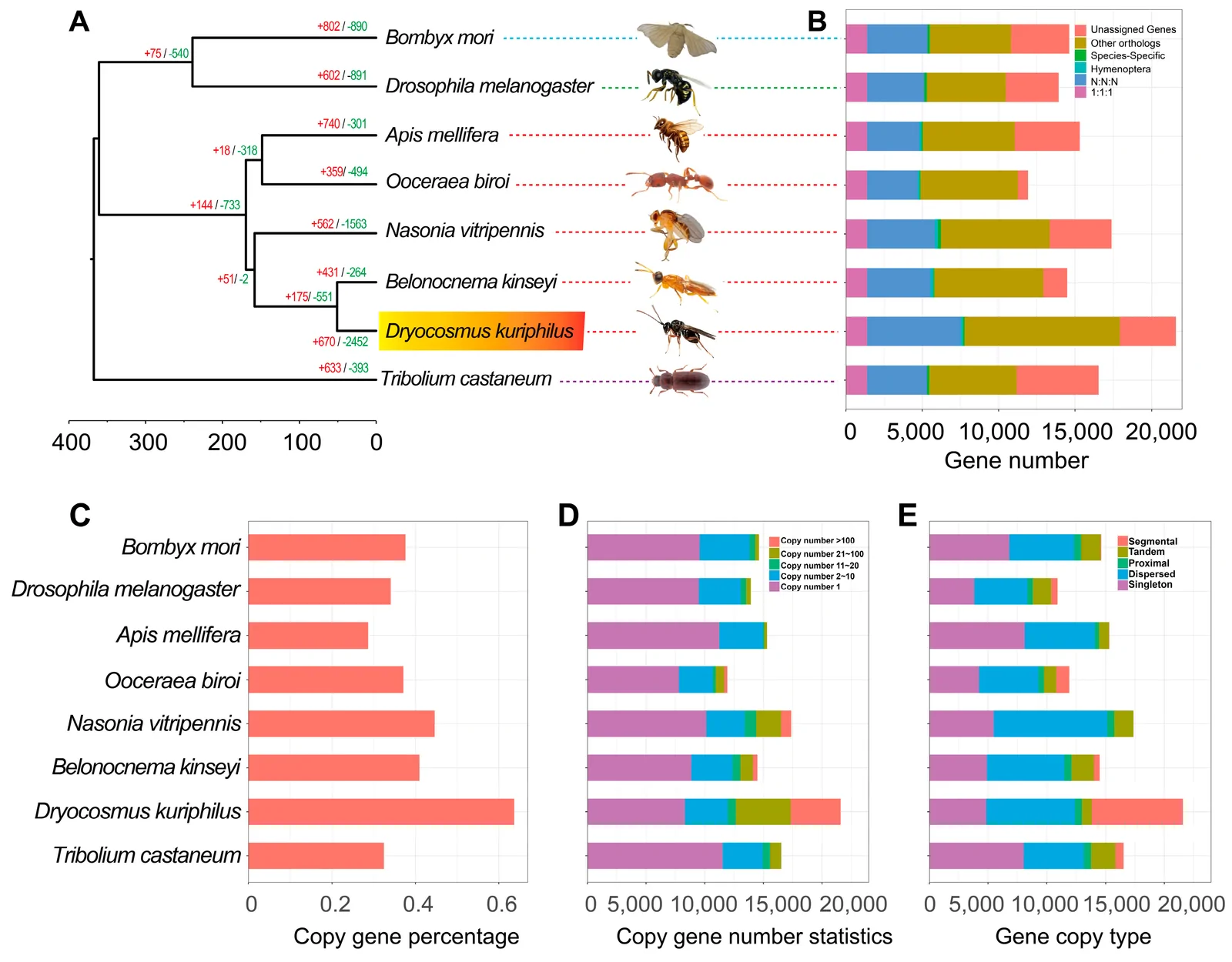
Genome and gene-family evolution in *Dryocosmus kuriphilus*. (**A**) Phylogeny of the eight species, with estimated divergence times and gene-family expansions and contractions. (**B**) Numbers of genes in different orthogroup categories. (**C**) Proportions of genes assigned to single-copy and multi-copy orthogroups by OrthoFinder. (**D**) Descriptive distribution of OrthoFinder-based within-species gene-family copy-number classes (single copy, 2–10 copies, 11–20 copies, 21–100 copies, and >100 copies). These copy-number classes were used for descriptive comparison only, and no inferential statistical test was applied. (**E**) Numbers of genes assigned to the five mutually exclusive MCScanX gene classes.

Orthologous relationships among the 8 genomes were inferred with the all-versus-all BLASTP strategy implemented in OrthoFinder. The analysis identified 37,024 orthogroups containing 125,759 protein-coding genes, including 1390 single-copy orthogroups. A phylogeny was reconstructed from the single-copy orthologues, with *Tribolium castaneum* as the outgroup, and divergence times were estimated on this framework (Figure 2A).

*Dryocosmus kuriphilus* clustered with *B. kinseyi* within Cynipidae, and their divergence was estimated at approximately 50 Ma (Figure 2A). This relationship provided the principal evolutionary comparison for subsequent analyses of repeat accumulation, genome expansion, and gene-family evolution.

Among the 37,024 orthogroups, 6129 contained multiple gene copies. Ninety-five orthogroups were restricted to the sampled hymenopterans, whereas 105 were specific to *D. kuriphilus* (Figure 2B). Within shared orthogroups displaying an N:N:N copy-number pattern, *D. kuriphilus* contributed approximately 30% more genes than the other species (Figure 2B), indicating elevated copy numbers within otherwise shared gene sets.

CAFE identified 5262 expanded and 9392 contracted gene families across the eight-species phylogeny. The *D. kuriphilus* branch contained 670 expanded and 2452 contracted families, compared with 431 expanded families in *B. kinseyi* (Figure 2A). The *D. kuriphilus* expansions were enriched for pathways associated with nutrient and amino-acid metabolism, detoxification and antioxidant defence, nucleic-acid metabolism, cellular homeostasis, immunity, and signal transduction. Among the 25 most enriched pathways, lysine degradation, tyrosine metabolism, arginine and proline metabolism, and cobalamin transport were associated with nutrient conversion. Glutathione metabolism and drug metabolism-other enzymes represented detoxification and oxidative-stress functions, whereas RNA degradation, mitophagy-animal, necroptosis, and cellular senescence represented RNA regulation, mitochondrial quality control, and cellular stress responses (Supplementary Figure S4).

Additional enriched pathways included nitrogen metabolism, alanine, aspartate and glutamate metabolism, cysteine and methionine metabolism, and amino-acid biosynthesis. ABC transporters, platinum-drug resistance, and antifolate resistance represented transport and xenobiotic-efflux functions. Immune-related categories included Toll and Imd, NOD-like receptor, cytosolic DNA-sensing, and NF-κB signalling, together with phagosome and lysosome pathways. AMPK, HIF-1, mTOR, insulin, and FoxO signalling represented nutrient sensing, growth regulation, hypoxia responses, and stress tolerance (Supplementary Table S2). Thus, the expanded *D. kuriphilus* gene families covered multiple functional systems relevant to nutrient use, detoxification, immunity, and cellular homeostasis.

CAFE therefore provided the phylogenetically informed assessment of lineage-specific gene-family expansion and contraction. We next summarized within-species orthogroup copy-number distributions descriptively and independently examined gene-duplication modes using MCScanX. The *D. kuriphilus* genome contained 21,584 protein-coding genes, 19.5–44.7% more than the seven comparison species (Table 2). OrthoFinder assigned 13,271 *D. kuriphilus* genes to multi-copy orthogroups, representing 61.5% of the protein-coding gene set. The >100-copy class contained 4251 genes distributed among 26 high-copy orthogroups. Thus, the large number of genes in the >100-copy class was concentrated in a relatively small number of orthogroups rather than broadly distributed across the gene-family repertoire. Using the same operational threshold, the other seven species contained 0–7 high-copy orthogroups (Supplementary Figure S8; Supplementary Table S5). These counts are presented descriptively and are not interpreted as evidence of lineage-specific gene-family expansion. Evolutionary expansion and contraction were evaluated independently using CAFE.

Independent MCScanX analysis assigned each *D. kuriphilus* gene to a single mutually exclusive duplication class. The final classification contained 4857 singleton genes, 7560 dispersed duplicates, 582 proximal duplicates, 818 tandem duplicates, and 7767 segmental duplicates (Figure 2E; Supplementary Table S6). Excluding singletons, 16,727 genes were classified as duplicated genes by MCScanX. Among these duplicated genes, segmental, dispersed, tandem, and proximal duplicates accounted for 46.43%, 45.20%, 4.89%, and 3.48%, respectively. Segmental duplicates represented the largest duplication class, followed by dispersed duplicates.

Ks distributions showed density peaks in lower and higher ranges. We describe these ranges as low-Ks and high-Ks components rather than assigning precise evolutionary periods. Without an explicit mixture model, a defined pair-selection procedure, and tests for Ks saturation, the current data do not establish duplication dates or prove that the high-Ks component predates divergence between the two cynipid species. The lower-Ks component contained 76.6% of dispersed, 86.1% of proximal, 85.6% of tandem, and 17.9% of Segmental duplicate pairs. The higher-Ks component was dominated by Segmental pairs and contained 48.1% of that duplication category.

These operational Ks components comprise duplicate pairs with contrasting levels of synonymous divergence. Functional composition was therefore compared between the two Ks components and among duplication modes.

**Figure 3:**
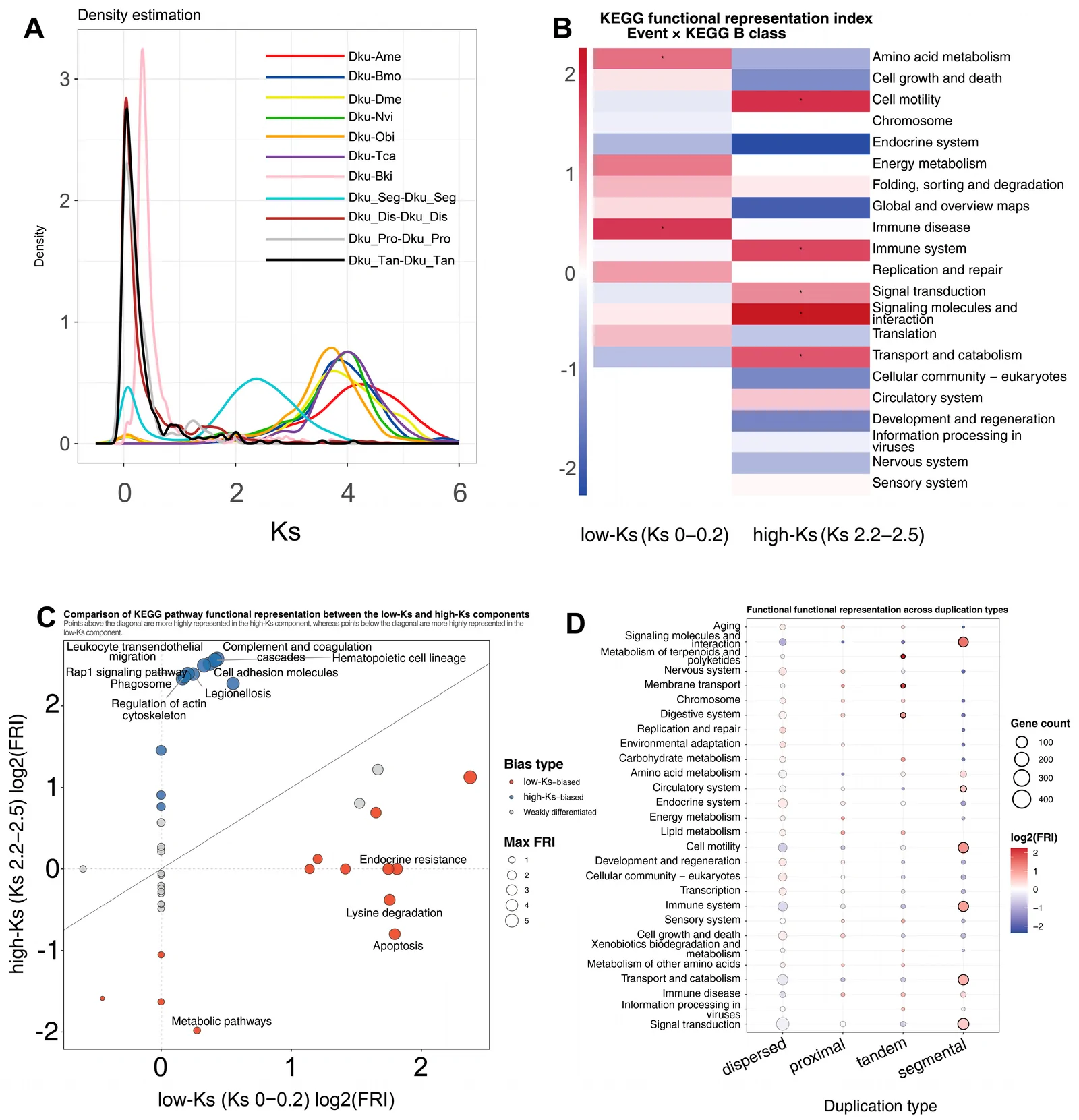
Patterns of gene duplication and functional representation in *D. kuriphilus*. (**A**) Kernel-density distributions of synonymous-substitution rates (Ks) for duplicate pairs in the indicated comparison groups. The low-Ks component was defined as Ks = 0–0.2 and the high-Ks component as Ks = 2.2–2.5, using symmetric intervals around the corresponding local density maxima. These intervals were used as operational subsets for exploratory comparison and do not represent discrete duplication events. (**B**) KEGG functional representation index (FRI) for broad functional classes in the low-Ks and high-Ks components. Colours indicate log2-transformed FRI values, with positive and negative values representing relative over-representation and under-representation, respectively. Asterisks indicate significant over-representation relative to the genomic reference background based on a one-sided hypergeometric test with Benjamini–Hochberg correction (adjusted *p* < 0.05). These significance markers do not represent direct statistical comparisons between the two Ks components. (**C**) Descriptive comparison of KEGG pathway representation between the low-Ks and high-Ks components. Points above and below the diagonal indicate greater relative representation in the high-Ks and low-Ks components, respectively. Point size represents the maximum FRI of the two components, and colour indicates low-Ks-biased, high-Ks-biased, or weakly differentiated representation based on differences in log2(FRI). These categories do not indicate statistically significant between-component differences. (**D**) Functional representation among dispersed, proximal, tandem, and segmental duplicates. Bubble size indicates gene number, and colour indicates log2-transformed FRI.

Genes in the low-Ks component showed greater relative representation of categories associated with amino-acid metabolism, RNA degradation, translation, and protein folding, whereas the high-Ks component showed greater relative representation of signal transduction, cell motility, transport, and cellular interactions (Figure 3B,C). The two components therefore differed in their functional composition.

Functional composition also differed among duplication modes. Segmental duplicates showed greater representation of genes associated with signal transduction and cellular processes, whereas tandem duplicates showed greater representation of transport, carbohydrate metabolism, and xenobiotic metabolism (Figure 3D). Thus, the duplicate groups differed in the relative representation of specific functional categories in *D. kuriphilus*.

### 3.3. TE-Associated Local Duplications Expand Adaptation-Related Gene Sets

Two structural patterns of TE-associated gene co-duplication were identified among OrthoFinder-defined duplicated genes. Type I comprised 841 events involving 1712 genes, in which the TE, the intervening sequence, and the duplicated gene all showed >95% nucleotide identity between duplicated loci, indicating co-duplication of a continuous TE-containing genomic segment. Type II comprised 53 events involving 172 genes, in which the TE and duplicated gene remained highly similar, but the intervening sequences were more divergent.

Subsequent classification of these duplicated genes using MCScanX showed that the majority of TE-associated co-duplication events belonged to the tandem-duplication category. This predominance indicates that tandem duplication was the most frequently represented duplication mode among the TE-containing co-duplicated segments, whereas dispersed and segmental duplications were less frequent. This descriptive pattern does not demonstrate that TE activity initiated the duplication event.

**Figure 4:**
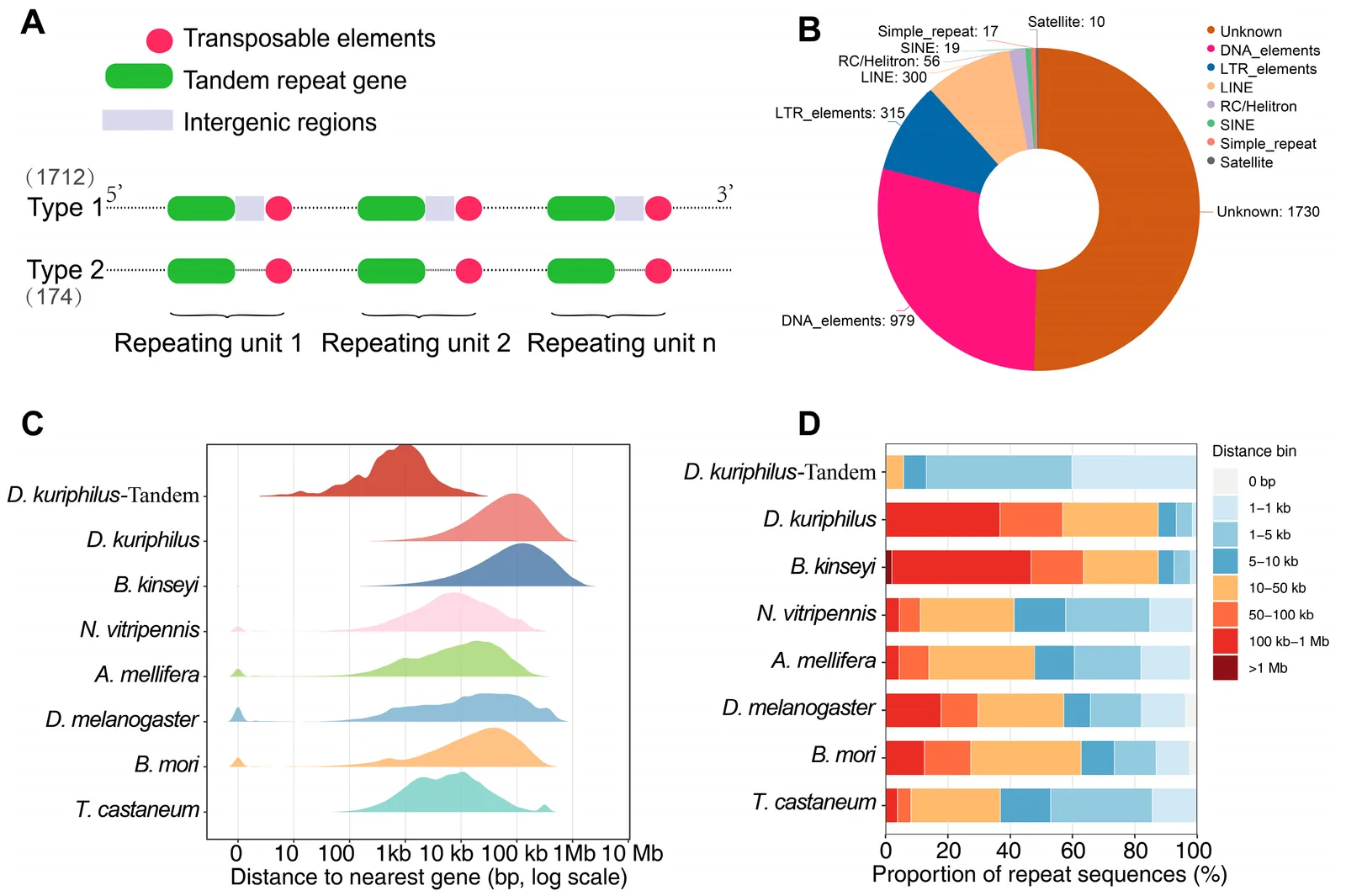
Spatial distribution of transposable elements relative to local gene duplications. (**A**) Two structural classes of TE-associated duplicated segments. Type 1 contains duplicated TE, intervening, and gene sequences, whereas Type 2 retains high similarity in the TE and gene sequences but lower similarity in the intervening sequence. (**B**) Classification of TEs associated with tandemly duplicated genes. (**C**) Distances between repeats and their nearest genes in TE-containing tandem-duplication regions and across the eight genomes, shown on a logarithmic scale. (**D**) Proportional distribution of repeats across distance intervals from the nearest gene, ranging from overlap (0 bp) to >1 Mb.

Among 3324 TEs upstream of tandemly duplicated genes, unclassified elements were most abundant at 50.5% (1730 sequences), followed by DNA transposons at 28.6% (979 sequences). LTR and LINE elements accounted for 315 and 300 sequences, respectively, and together represented 17.9% (Figure 4B). TEs in these tandem-duplication-associated regions showed a distance peak at approximately 600 bp from the genes (Figure 4C). More than 85% of these repeats occurred within 0–5 kb of a gene, whereas genome-wide repeats in *D. kuriphilus* and the other species more frequently occurred at distances of at least 10 kb (Figure 4D). Thus, TEs in these co-duplicated regions were frequently located within 5 kb of tandemly duplicated genes. Because no permutation or randomization test against a genomic null expectation was performed, this pattern is interpreted descriptively rather than as evidence of preferential spatial association.

Among genes in TE-associated tandem duplications, 166 were assigned to three adaptation-related categories: 66 to feeding or host use, 63 to detoxification, and 37 to energy metabolism (Supplementary Figure S5; Supplementary Table S3). The feeding category included olfactory receptors, proteases, amino-acid and metal-ion transporters, and nutrient-absorption receptors. The detoxification category included genes involved in phase I oxidation, phase II conjugation, reactive-oxygen-species metabolism, and ABC, MFS, or SLC transport. Energy-metabolism genes represented the tricarboxylic acid cycle, mitochondrial function, oxidative phosphorylation, lipid metabolism, and adenylate-energy conversion. TE-associated tandem duplicates included genes annotated with functions related to host interaction, xenobiotic processing, and energy production. Functional enrichment analysis of the TE-associated tandem duplicated genes, using all annotated *D. kuriphilus* genes as the genomic background, revealed significant over-representation of multiple KEGG pathways related to metabolism, immune functions, and signal transduction (Supplementary Figure S9A,B).

GO enrichment analysis further showed significant enrichment of biological-process terms including response to stimulus, immune system process, biological adhesion, and rhythmic process (Supplementary Figure S9C). Together, these results indicate that TE-associated tandem duplicated genes are non-randomly distributed across functional categories, with significant over-representation of categories related to metabolism, immunity, signalling, and environmental responses.

### 3.4. Copy-Number Differences in Candidate Gene Families Associated with Host Use and Environmental Responses

**Figure 5:**
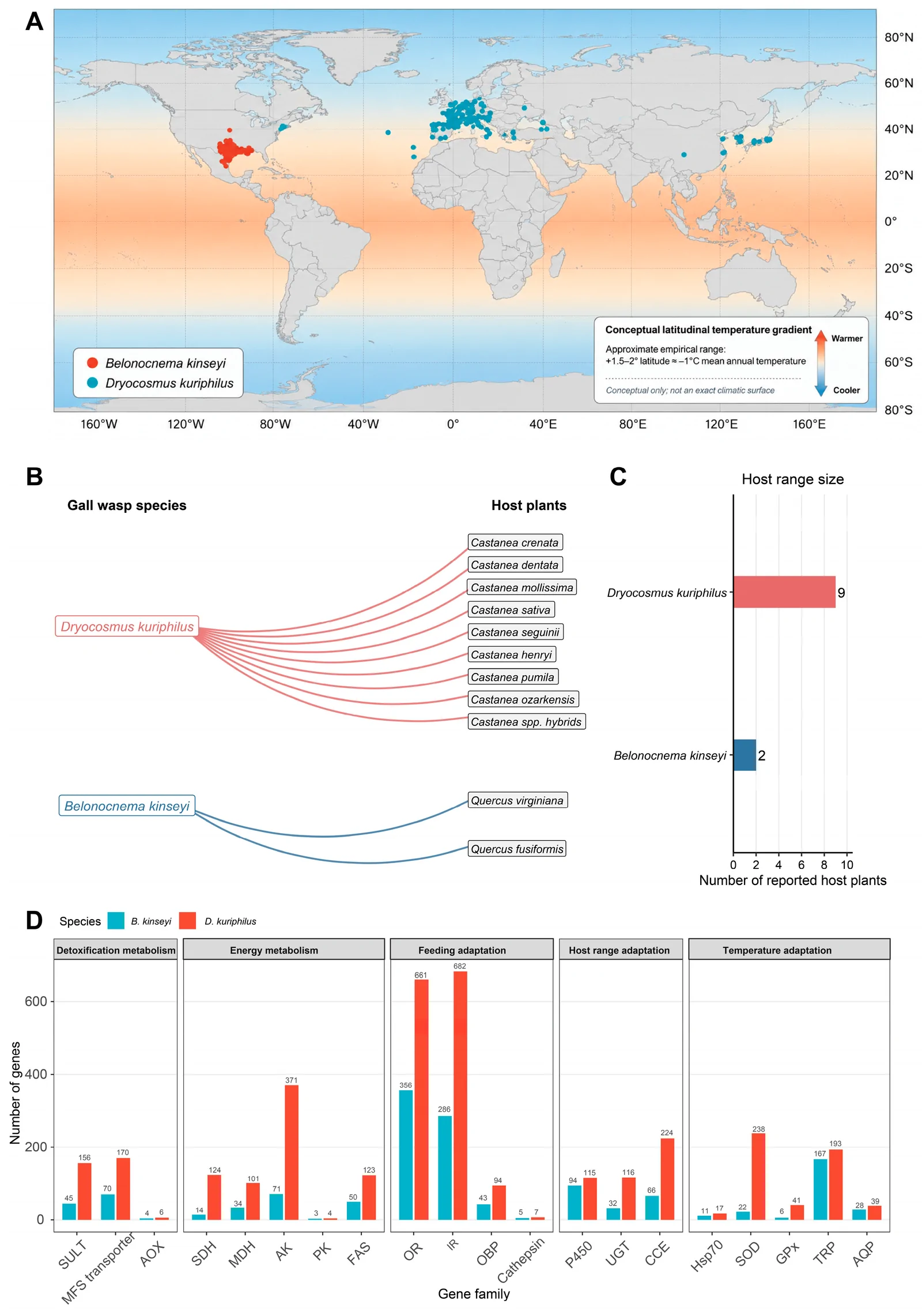
Ecological distributions, host ranges, and gene-family differences between *D. kuriphilus* and *B. kinseyi*. (**A**) GBIF occurrence records, with *D. kuriphilus* in blue and *B. kinseyi* in red. Background shading represents a conceptual latitudinal temperature gradient. (**B**) Reported host associations. (**C**) Numbers of documented host plants. (**D**) Copy numbers of selected families associated with detoxification, energy metabolism, feeding, chemosensation, and stress responses. Abbreviations: SULT, sulfotransferase; MFS, major facilitator superfamily transporter; AOX, aldehyde oxidase; SDH, succinate dehydrogenase; MDH, malate dehydrogenase; AK, adenylate kinase; PK, pyruvate kinase; FAS, fatty acid synthase; OR, odorant receptor; IR, ionotropic receptor; OBP, odorant-binding protein; P450, cytochrome P450 monooxygenase; UGT, UDP-glycosyltransferase; CCE, carboxyl/cholinesterase; Hsp70, heat-shock protein 70; SOD, superoxide dismutase; GPX, glutathione peroxidase; TRP, transient receptor potential channel; AQP, aquaporin.

GBIF (https://www.gbif.org/citation-guidelines (accessed on 16 August 2026)) occurrence records indicated a substantially broader reported longitudinal distribution for *D. kuriphilus* (−77.076256° to 140.099040°) than for *B. kinseyi* (−102.1082° to −87.935036°; Figure 5A). Reported host associations of *B. kinseyi* were largely confined to Quercus virginiana and *Q. fusiformis*, whereas *D. kuriphilus* was recorded from eight *Castanea* species and their hybrids (Figure 5B,C; Supplementary Table S4). These patterns provide a descriptive comparison of the reported ecological breadth of the two species but should not be interpreted as evidence that variation in gene-family size directly underlies the broader geographic or host range observed for *D. kuriphilus*. Occurrence and host records may be influenced by differences in sampling intensity, host availability, invasion history, and human-mediated dispersal. Consistent with the copy-number patterns described above, *D. kuriphilus* exhibited higher copy numbers than *B. kinseyi* in several candidate gene families associated with nutrient acquisition, detoxification, immune function, energy homeostasis, and responses to environmental stress (Supplementary Figure S6). However, these copy-number differences alone are insufficient to establish lineage-specific evolutionary expansion or a causal relationship with ecological breadth.

Genes present at higher copy numbers in *D. kuriphilus* than in *B. kinseyi* included ORs, IRs, and OBPs associated with chemosensory perception; P450s, UGTs, CCEs, SULTs, MFS transporters, and AOXs associated with xenobiotic metabolism and transport; SDHs, MDHs, AKs, PKs, and FASs associated with energy metabolism; and Hsp70s, SODs, GPXs, TRPs, and AQPs associated with cellular stress responses, temperature sensing, and water balance (Figure 5D). These copy-number differences were observed in candidate gene families associated with host recognition, xenobiotic metabolism, energy metabolism, and environmental responses.

Genes with higher copy numbers in *D. kuriphilus* than in *B. kinseyi*, including OR, IR, AK, SOD, CCE, UGT, and MFS transporter families, were frequently assigned to the segmental duplication class. TE-associated tandem duplication contributed additional copies of genes involved in host recognition, detoxification, and energy metabolism (Supplementary Figure S7). Thus, large-scale and local duplication processes contributed complementary components to the higher-copy gene repertoire of *D. kuriphilus*.

## 4. Discussion

Comparative genomic analyses identified three principal features of the *D. kuriphilus* genome: extensive repeat accumulation, elevated gene copy number, and functional differences among duplicate groups. *D. kuriphilus* was most closely related to *B. kinseyi*, with an estimated divergence time of approximately 50 Ma. It contained more genes in several shared orthogroups, while the CAFE analysis identified 670 expanded gene families along the *D. kuriphilus* branch compared with 431 in *B. kinseyi*. These CAFE-inferred expansions involved functional categories related to metabolism, signalling, immunity, digestion, and responses to external stimuli.

Gene-family expansion may nevertheless provide a mechanistic basis for generating phenotypic variation. Gene duplication can increase gene dosage and, for dosage-sensitive genes, may increase transcript or protein abundance, while duplicated copies can also subsequently undergo regulatory or functional divergence [52,53]. However, the relationship between copy number and expression is not universal, as dosage compensation or regulatory effects can buffer transcriptional responses to copy-number changes [54]. In insects, a well-characterized example is the amplification of CYP6CY3 in tobacco-adapted Myzus persicae, where increased copy number contributes to elevated expression, enhanced nicotine detoxification, and host adaptation [55]. Thus, the higher-copy chemosensory, detoxification, metabolic, and stress-response families identified in *D. kuriphilus* may provide increased gene dosage or additional substrates for functional diversification, but their contribution to ecological adaptation remains a hypothesis requiring expression and functional validation.

Similar candidate relationships between gene-family expansion and ecological breadth have been reported in other herbivorous insects. Spodoptera frugiperda shows expansions of chemosensory and detoxification genes, many generated through tandem duplication, whereas highly polyphagous Helicoverpa species possess expanded detoxification, digestion, and chemosensory repertoires together with divergent expression responses to different host plants [56,57]. However, broader comparative analyses indicate that the association between gene-family size and polyphagy is not universal across all gene families or lepidopteran lineages [58].

Genome-size variation within Cynipoidea is broader than suggested by genome assemblies available from InsectBase alone. In particular, *Ganaspis brasiliensis* and *G. xanthopoda* (Figitidae) have genome sizes of approximately 831 and 972 Mb, respectively, approaching the lower range observed in Cynipini. These observations indicate that relatively large genomes have evolved in multiple cynipoid lineages and are not unique to Cynipini. However, current genome-size estimates remain taxonomically sparse and were generated using different approaches, so broader sampling will be required to determine the evolutionary pattern of genome-size change across Cynipoidea. The unusually large *D. kuriphilus* genome was closely associated with its high repeat content. Most TEs occurred in intergenic regions, and the two cynipid genomes combined extensive repeat accumulation with markedly elongated intergenic regions. These observations indicate that much of the additional genomic sequence in the sampled cynipids is located in repeat-rich, non-coding regions rather than reflecting proportional enlargement of coding regions. However, because the present comparative data do not reconstruct ancestral genome sizes or the temporal dynamics of repeat proliferation, they do not establish repeat accumulation as the evolutionary cause of genome expansion. Transposon activity can reshape genomic and cis-regulatory architecture, and repeat-rich regions may provide substrates for rearrangement and duplication, but the direction and timing of these processes remain unresolved.

Gene-family size variation was another prominent feature of the *D. kuriphilus* genome. OrthoFinder assigned 13,271 genes to multi-copy orthogroups, and 4251 genes in the >100-copy class were concentrated in only 26 high-copy orthogroups. This pattern indicates that the upper end of the within-species copy-number distribution was driven by a relatively small subset of orthogroups rather than by uniformly high copy numbers across gene families. However, high absolute copy number does not by itself demonstrate lineage-specific evolutionary expansion. Accordingly, the OrthoFinder copy-number classes are interpreted descriptively, whereas evolutionary gene-family expansion and contraction are inferred from the phylogenetically informed CAFE analysis. CAFE identified 670 expanded and 2452 contracted gene families along the *D. kuriphilus* branch.

Independent MCScanX analysis further showed that duplicated genes were distributed among mutually exclusive segmental, dispersed, tandem, and proximal classes. Among the MCScanX-classified duplicated genes, segmental and dispersed were the predominant classes. These results describe the genomic organization of duplicated genes but should not be interpreted as implying that the MCScanX classes account directly for the 13,271 genes identified in the OrthoFinder multi-copy analysis. The lower- and higher-Ks components represent operational subsets of duplicated gene pairs with different levels of synonymous divergence and do not establish the timing of duplication events relative to the divergence between *D. kuriphilus* and *B. kinseyi*. Their distinct functional profiles indicate differences in the relative representation of functional categories among duplicate groups. Regulatory and cellular-process categories were more strongly represented among segmental duplicates, whereas transport and xenobiotic-metabolism categories were more strongly represented among tandem duplicates. These patterns describe the functional composition of extant duplicate groups but do not establish preferential evolutionary retention or differential loss from an ancestral gene repertoire.

Many repeats in TE-containing tandem-duplication regions occurred within 5 kb of tandemly duplicated genes, and two classes of high-similarity duplicated segments were detected. These observations describe genomic proximity but do not establish preferential spatial association because no genomic null model or randomization test was applied. TE-associated tandem duplicates and segmental duplicates overlapped in KEGG categories related to energy metabolism, transmembrane transport, transcriptional regulation, DNA repair, and environmental interactions. Expanded P450 and IR families further linked both duplication modes to xenobiotic metabolism and chemosensation. These associations support a model in which TEs and segmental duplication contribute at different genomic scales, although direct TE causation cannot be inferred from sequence proximity alone.

Together, the results support an association model rather than a demonstrated temporal sequence of genome evolution in *D. kuriphilus*. Repeat-rich architecture co-occurs with expanded intergenic regions, while several duplication classes co-occur with increased gene copy number and distinct functional profiles. The available comparative data do not establish whether repeat proliferation preceded genome expansion, whether TEs directly caused gene duplications, or whether the observed genomic expansions were adaptive. These relationships therefore provide hypotheses for future phylogenetic, population-genomic, and functional analyses rather than a resolved sequence of evolutionary events.

Across both large-scale and local contexts, multiple duplication mechanisms converged on gene sets associated with host use and environmental responses. Compared with B. kinseyi, *D. kuriphilus* had a broader reported distribution and host range and contained more ORs, IRs, OBPs, P450s, UGTs, CCEs, SULTs, MFS transporters, metabolic enzymes, and stress-response genes. Related genomic studies have implicated changes in detoxification, energy metabolism, and stress-response genes in insect adaptation to environmental challenges [39,59]. Many genes from these higher-copy candidate families were assigned to the segmental duplication class, whereas TE-associated local duplication included additional copies of genes involved in host recognition, detoxification, and energy metabolism. These differences do not show that a larger gene repertoire caused invasion success or host adaptation. They identify candidate genomic differences for future tests of selection, expression, functional divergence, and population-level association, while observed host and geographic ranges remain influenced by sampling effort, host availability, and human-mediated dispersal. Compared with *B. kinseyi*, *D. kuriphilus* contained more genes in several families associated with chemosensation, detoxification, metabolism, and stress responses. Increased copy number may potentially increase gene dosage in dosage-sensitive loci or provide additional genetic substrates for regulatory and functional divergence. Similar mechanisms have contributed to host or environmental responses in other insects [55,56,57]. However, the present data do not demonstrate that these expansions caused the broader reported host range, geographic distribution, or invasion success of *D. kuriphilus*. Instead, they identify candidate genomic differences for future tests using transcriptomics, tests of selection, biochemical assays, and population-level association analyses.

These findings also provide a cautious framework for considering genome evolution under thelytoky. The genomic consequences of thelytokous reproduction depend on its underlying cytological mechanism, because apomictic and different automictic pathways can have distinct effects on recombination and the maintenance of heterozygosity [26]. Therefore, thelytoky in *D. kuriphilus* should not be interpreted simply as a complete absence of recombination. Nevertheless, compared with conventional sexual reproduction, thelytoky generally provides fewer opportunities for allelic reshuffling, potentially constraining the generation of genetic variation available for long-term adaptation. In this context, the high repeat content, numerous collinear duplicates, TE-associated local duplications, and high-copy gene families observed in *D. kuriphilus* are consistent with structural and copy-number variation as additional sources of genomic diversity. However, these associations do not demonstrate that gene duplication or TE activity compensates for reduced sexual recombination. In Timema stick insects, parthenogenesis is associated with reduced heterozygosity and adaptive efficiency without a general increase in TE abundance, indicating that asexual lineages can follow different genomic trajectories [60].

This study has several limitations. The conclusions are based primarily on comparative genomics and functional annotation, and direct experimental evidence for the roles of expanded families and TE-associated duplications is lacking. Observed differences in geographic and host ranges are also influenced by sampling effort, host availability, and human-mediated dispersal. Integrating transcriptomics, chromatin-accessibility profiling, population genomics, and functional experiments will be necessary to test the roles of candidate genes and TEs in host use, detoxification, immunity, and environmental responses.

## Figures and Tables

**Table 1:** Genome assemblies used for comparative analysis.

Order	Family	Species	Accession Number
Hymenoptera	Cynipidae	*Dryocosmus kuriphilus*	GCA_038277395.1
Hymenoptera	Formicidae	*Ooceraea biroi*	GCF_003672135.2
Hymenoptera	Apidae	*Apis mellifera*	GCF_003254395.2
Hymenoptera	Cynipidae	*Belonocnema kinseyi*	GCA_010883055.1
Hymenoptera	Pteromalidae	*Nasonia vitripennis*	GCF_009193385.2
Diptera	Drosophilidae	*Drosophila melanogaster*	GCF_000001215.4
Lepidoptera	Bombycidae	*Bombyx mori*	GCF_030269925.1
Coleoptera	Tenebrionidae	*Tribolium castaneum*	GCF_031307605.1

**Table 2:** Genome sizes and protein-coding gene numbers for the eight species.

Species	Number of Genes	Genome Size (bp)
*Nasonia vitripennis*	17,370	295,085,711
*Ooceraea biroi*	11,927	50,585,078
*Apis mellifera*	15,314	234,070,657
*Belonocnema kinseyi*	14,488	1,538,686,255
*Drosophila melanogaster*	13,929	143,727,871
*Bombyx mori*	14,623	480,775,871
*Tribolium castaneum*	16,524	228,746,138
*Dryocosmus kuriphilus*	21,584	2,283,620,086

**Table 3:** Repeat annotation of the *Dryocosmus kuriphilus* genome.

Transposable-Element Class	Length (Mb)	Genome Proportion (%)
SINEs	8.3	0.4
MIRs	1.5	0.1
LINEs	223.3	9.8
LINE1	5.7	0.02
LINE2	57.2	0.3
L3/CR1	30.5	0.1
LTR elements	148.7	6.5
ERV_classI	3.0	0.1
ERV_classII	0.2	0.01
DNA elements	461.9	20.3
Unclassified	587.1	25.8
Total TEs	1419.4	62.3
Tandem Repeats	396.5	17.4
Total Repeats	1815.8	79.7

**Table 4:** Transposable-element content of the 8 insect genomes.

Species	Transposable-Element Length (Mb)	Genome Proportion (%)
*Dryocosmus kuriphilus*	1419.4	62.3
*Ooceraea biroi*	38.02	17.0
*Apis mellifera*	11.5	4.9
*Belonocnema kinseyi*	1002.3	65.1
*Nasonia vitripennis*	66.0	22.4
*Drosophila melanogaster*	28.1	19.5
*Bombyx mori*	184.1	38.3
*Tribolium castaneum*	42.8	18.7

## Data Availability

The original contributions presented in this study are included in the article. Further inquiries can be directed to the corresponding authors.

## Supplementary Materials

The following supporting information can be downloaded at https://www.mdpi.com/article/10.3390/insects17101047/s1. Figure S1: Comparison of genome sizes among Cynipini, non-Cynipini, and other hymenopteran families. Figure S2: Relative composition of repetitive sequence types in the genomes of eight insect species. Figure S3: Repeat composition in the intergenic regions of *Dryocosmus kuriphilus* and *Belonocnema kinseyi*. (A) Repeat composition in the intergenic regions of *D. kuriphilus*. (B) Repeat composition in the intergenic regions of *B. kinseyi*. Figure S4: KEGG pathway enrichment analysis of candidate genes. Figure S5: Numbers of genes associated with feeding adaptation, detoxification metabolism, and energy metabolism. Figure S6: KEGG pathway annotation and functional classification of candidate genes. Figure S7: Distribution of gene duplication types among different functional gene families. Figure S8: Numbers of high-copy orthogroups (>100 within-species gene copies) across the eight insect species. Figure S9: Functional enrichment of genes associated with transposon-associated tandem duplication in the *D. kuriphilus* genome. (A) KEGG enrichment of genes at Level 3 (*p* < 0.01). (B) KEGG functional classification of genes at Level 2 (*p* < 0.01). (C) GO enrichment analysis of the genes (*p* < 0.01). Table S1: Genome sizes of 413 hymenopteran species and their data sources. Table S2: KEGG pathway enrichment analysis of transposon-associated tandemly duplicated genes in the *D. kuriphilus* genome. Table S3: Functional annotation and categorization of selected genes from families with higher copy numbers in *D. kuriphilus*. Table S4: Reported host plants of *D. kuriphilus* and *B. kinseyi*. Table S5: High-copy orthogroups containing >100 within-species gene copies across the eight insect species. Table S6: Numbers of genes assigned to different duplication categories across the eight insect species. Table S7: BUSCO completeness and gene-model statistics for the eight insect species used in comparative genomic analyses.

## References

[1] Abe Y., Melika G., Stone G.N. (2007). The diversity and phylogeography of cynipid gallwasps (Hymenoptera: Cynipidae) of the Oriental and eastern Palearctic regions, and their associated communities. Orient. Insects.

[2] Aebi A., Schönrogge K., Melika G., Quacchia A., Alma A., Stone G.N. (2007). Native and introduced parasitoids attacking the invasive chestnut gall wasp *Dryocosmus kuriphilus*. EPPO Bull..

[3] EPPO (2005). *Dryocosmus* *kuriphilus*. EPPO Bull..

[4] Quacchia A., Moriya S., Bosio G., Scapin I., Alma A. (2008). Rearing, release and settlement prospect in Italy of Torymus sinensis, the biological control agent of the chestnut gall wasp *Dryocosmus kuriphilus*. BioControl.

[5] EPPO *Dryocosmus kuriphilus* (DRYCKU). EPPO Global Database. https://gd.eppo.int/taxon/DRYCKU.

[6] Graziosi I., Santi F. (2008). Chestnut gall wasp (*Dryocosmus kuriphilus*): Spreading in Italy and new records in Bologna province. Bull. Insectol..

[7] Huber J.T., Read J. (2012). First record of the oriental chestnut gall wasp, *Dryocosmus kuriphilus* Yasumatsu (Hymenoptera: Cynipidae), in Canada. J. Entomol. Soc. Ont..

[8] Rieske L.K. (2007). Success of an exotic gallmaker, *Dryocosmus kuriphilus*, on chestnut in the USA: A historical account. EPPO Bull..

[9] Shiraga T. (1951). Problems and control of the chestnut gall wasp. Nōgyō Oyobi Engei (Agric. Hortic.).

[10] Graziosi I., Rieske L.K. (2014). Potential fecundity of a highly invasive gall maker *Dryocosmus kuriphilus* (Hymenoptera, Cynipidae). Environ. Entomol..

[11] Zhu D., He Y., Fan Y., Ma M., Peng D. (2007). Negative evidence of parthenogenesis induction by *Wolbachia* in a gallwasp species, *Dryocosmus kuriphilus*. Entomol. Exp. Appl..

[12] Gilioli G., Pasquali S., Tramontini S., Riolo F. (2013). Modelling local and long-distance dispersal of invasive chestnut gall wasp in Europe. Ecol. Model..

[13] Battisti A., Benvegnù I., Colombari F., Haack R.A. (2014). Invasion by the chestnut gall wasp in Italy causes significant yield loss in *Castanea sativa* nut production. Agric. For. Entomol..

[14] Kato K., Hijii N. (1997). Effects of gall formation by *Dryocosmus kuriphilus* Yasumatsu (Hym, Cynipidae) on the growth of chestnut trees. J. Appl. Entomol..

[15] Sartor C., Dini F., Torello Marinoni D., Mellano M., Beccaro G., Alma A., Quacchia A., Botta R. (2015). Impact of the Asian wasp *Dryocosmus kuriphilus* (Yasumatsu) on cultivated chestnut: Yield loss and cultivar susceptibility. Sci. Hortic..

[16] EFSA Panel on Plant Health (PLH) (2010). Risk assessment of the oriental chestnut gall wasp, *Dryocosmus kuriphilus*, for the EU territory and identification and evaluation of risk management options. EFSA J..

[17] Fernandes P., Serrazina S., Pavese V., Martín M.A., Mattioni C., Martínez M., Piñeiro P., Fraga M., Cuenca B., Moglia A. (2026). Biotechnological advances for enhancing European chestnut resistance to pests, diseases, and climate change. Horticulturae.

[18] Lobo Santos A., Santos S.A.P., Casquero P.A., Bento A. (2024). Lifecycle of *Dryocosmus kuriphilus* Yasumatsu and diversity and importance of the native parasitoid community recruited in northern Portugal. Insects.

[19] Martinez-Sañudo I., Mazzon L., Simonato M., Avtzis D., Pujade-Villar J., Faccoli M. (2019). Tracking the origin and dispersal of the Asian chestnut gall wasp *Dryocosmus kuriphilus* Yasumatsu (Hymenoptera, Cynipidae) in Europe with molecular markers. Bull. Entomol. Res..

[20] Quinto J., Wong M.E., Boyero J.R., Vela J.M., Aguirrebengoa M. (2021). Population dynamics and tree damage of the invasive chestnut gall wasp, *D. kuriphilus*, in its southernmost European distributional range. Insects.

[21] Romay-Río M.F., Castedo-Dorado F., Ayres M.P., Prado A.N., Lombardero M.J. (2024). Interactions between invasive pests and pathogens in a native chestnut forest. NeoBiota.

[22] Gokhman V.E., Ilinsky Y.Y., Kuznetsova V.G. (2026). Parthenogenesis and sex determination in the order Hymenoptera (Insecta): Patterns and mechanisms. Entomol. Exp. Appl..

[23] Lacy K.D., Hart T., Kronauer D.J.C. (2024). Co-inheritance of recombined chromatids maintains heterozygosity in a parthenogenetic ant. Nat. Ecol. Evol..

[24] Villegas L.I., Ferretti L., Wiehe T., Waldvogel A., Schiffer P.H. (2024). Parthenogenomics: Insights on mutation rates and nucleotide diversity in parthenogenetic *Panagrolaimus nematodes*. Ecol. Evol..

[25] Parée T., Teotónio H. (2025). Experimental tests on the evolution of sex and recombination and their adaptive significance. J. Evol. Biol..

[26] Tichkule S., van Oosterhout C., Cacciò S.M., Weisz F., Balan B., Nohýnková E., Naung M., Ansell B.R.E., Emery-Corbin S.J., Baker L. (2025). Host range expansion of asexual parasite can be explained by loss of adaptions in Muller’s Ratchet. Nat. Commun..

[27] Warren R.J., Mokadam C. (2025). Asexuality and species invasion. Biodivers. Conserv..

[28] Assis R., Conant G., Holland B., Liberles D.A., O’Reilly M.M., Wilson A.E. (2024). Models for the retention of duplicate genes and their biological underpinnings. F1000Research.

[29] Betancourt A.J., Wei K.H.-C., Huang Y., Lee Y.C.G. (2024). Causes and consequences of varying transposable element activity: An evolutionary perspective. Annu. Rev. Genom. Hum. Genet..

[30] Birchler J.A., Yang H. (2022). The multiple fates of gene duplications: Deletion, hypofunctionalization, subfunctionalization, neofunctionalization, dosage balance constraints, and neutral variation. Plant Cell.

[31] Garber A.I., Sano E.B., Gallagher A.L., Miller S.R. (2024). Duplicate gene expression and possible mechanisms of paralog retention during bacterial genome expansion. Genome Biol. Evol..

[32] Kuzmin E., Taylor J.S., Boone C. (2022). Retention of duplicated genes in evolution. Trends Genet..

[33] Wilson A.E., Liberles D.A. (2023). Dosage balance acts as a time-dependent selective barrier to subfunctionalization. BMC Ecol. Evol..

[34] Balachandran P., Walawalkar I.A., Flores J.I., Dayton J.N., Audano P.A., Beck C.R. (2022). Transposable element-mediated rearrangements are prevalent in human genomes. Nat. Commun..

[35] Emmerson R., Catoni M. (2025). The role of mobile DNA elements in the dynamics of plant genome plasticity. J. Exp. Bot..

[36] Kojima S. (2024). Investigating mobile element variations by statistical genetics. Hum. Genome Var..

[37] Ffrench-Constant R.H. (2023). Transposable elements and xenobiotic resistance. Front. Insect Sci..

[38] Sun Z., Chen Y., Chen Y., Lu Z., Gui F. (2023). Tracking adaptive pathways of invasive insects: Novel insight from genomics. Int. J. Mol. Sci..

[39] Weng Y.-M., Shashank P.R., Godfrey R.K., Plotkin D., Parker B.M., Wist T., Kawahara A.Y. (2024). Evolutionary genomics of three agricultural pest moths reveals rapid evolution of host adaptation and immune-related genes. GigaScience.

[40] Liu B., Ren Y.-S., Su C.-Y., Wang X.-D., Zeng Y., Zhu D.-H. (2024). Chromosome-level genome assembly of the Oriental chestnut gall wasp (*Dryocosmus kuriphilus*). Sci. Data.

[41] Emms D.M., Kelly S. (2015). OrthoFinder: Solving fundamental biases in whole genome comparisons dramatically improves orthogroup inference accuracy. Genome Biol..

[42] Wang Y., Tang H., DeBarry J.D., Tan X., Li J., Wang X., Lee T.-H., Jin H., Marler B., Guo H. (2012). MCScanX: A toolkit for detection and evolutionary analysis of gene synteny and collinearity. Nucleic Acids Res..

[43] Edgar R.C. (2004). MUSCLE: Multiple sequence alignment with high accuracy and high throughput. Nucleic Acids Res..

[44] Guindon S., Dufayard J.-F., Lefort V., Anisimova M., Hordijk W., Gascuel O. (2010). New algorithms and methods to estimate maximum-likelihood phylogenies: Assessing the performance of PhyML 3.0. Syst. Biol..

[45] Yang Z. (2007). PAML 4: Phylogenetic analysis by maximum likelihood. Mol. Biol. Evol..

[46] De Bie T., Cristianini N., Demuth J.P., Hahn M.W. (2006). CAFE: A computational tool for the study of gene family evolution. Bioinformatics.

[47] Benson G. (1999). Tandem repeats finder: A program to analyze DNA sequences. Nucleic Acids Res..

[48] Smit A.F.A., Hubley R. RepeatModeler Open-1.0. 2008–2015. http://www.repeatmasker.org/RepeatModeler/.

[49] Smit A.F.A., Hubley R., Green P. RepeatMasker Open-4.0. 2013–2015. http://www.repeatmasker.org/.

[50] Wang D., Zhang Y., Zhang Z., Zhu J., Yu J. (2010). KaKs_Calculator 2.0: A toolkit incorporating gamma-series methods and sliding window strategies. Genom. Proteom. Bioinform..

[51] Brandão-Dias P.F.P., Zhang Y.M., Pirro S., Vinson C.C., Weinersmith K.L., Ward A.K.G., Forbes A.A., Egan S.P. (2022). Describing biodiversity in the genomics era: A new species of Nearctic cynipid gall wasp and its genome. Syst. Entomol..

[52] Innan H., Kondrashov F. (2010). The evolution of gene duplications: Classifying and distinguishing between models. Nat. Rev. Genet..

[53] Kondrashov F.A. (2012). Gene duplication as a mechanism of genomic adaptation to a changing environment. Proc. R. Soc. B Biol. Sci..

[54] Zhou J., Lemos B., Dopman E.B., Hartl D.L. (2011). Copy-number variation: The balance between gene dosage and expression in Drosophila melanogaster. Genome Biol. Evol..

[55] Bass C., Zimmer C.T., Riveron J.M., Wilding C.S., Wondji C.S., Kaussmann M., Field L.M., Williamson M.S., Nauen R. (2013). Gene amplification and microsatellite polymorphism underlie a recent insect host shift. Proc. Natl. Acad. Sci. USA.

[56] Gouin A., Bretaudeau A., Nam K., Gimenez S., Aury J.-M., Duvic B., Hilliou F., Durand N., Montagné N., Darboux I. (2017). Two genomes of highly polyphagous lepidopteran pests (*Spodoptera frugiperda*) with different host-plant ranges. Sci. Rep..

[57] Pearce S.L., Clarke D.F., East P.D., Elfekih S., Gordon K.H.J., Jermiin L.S., McGaughran A., Oakeshott J.G., Papanikolaou A., Perera O.P. (2017). Genomic innovations, transcriptional plasticity and gene loss underlying the evolution and divergence of two highly polyphagous and invasive Helicoverpa pest species. BMC Biol..

[58] Breeschoten T., van der Linden C.F.H., Ros V.I.D., Schranz M.E., Simon S. (2022). Expanding the menu: Are polyphagy and gene family expansions linked across Lepidoptera?. Genome Biol. Evol..

[59] Wang Z., Liu Y., Wang H., Roy A., Liu H., Han F., Zhang X., Lu Q. (2023). Genome and transcriptome of Ips nitidus provide insights into high-altitude hypoxia adaptation and symbiosis. iScience.

[60] Jaron K.S., Parker D.J., Anselmetti Y., Tran Van P., Bast J., Dumas Z., Figuet E., François C.M., Hayward K., Rossier V. (2022). Convergent consequences of parthenogenesis on stick insect genomes. Sci. Adv..

