# Supplementary material for: Repeat-Rich Architecture and Gene Duplication Characterize the Expanded Genome of the Chestnut Gall Wasp
Source: Insects. 2026 Oct 12;17(10):1047. doi: 10.3390/insects17101047 (PMC13704927; doi:10.3390/insects17101047)
Supplement: Supplementary file 1 [file insects-17-01047-s001.zip › Supplementary_Fig.pdf]

Supplementary Material

1. Supplementary Figures

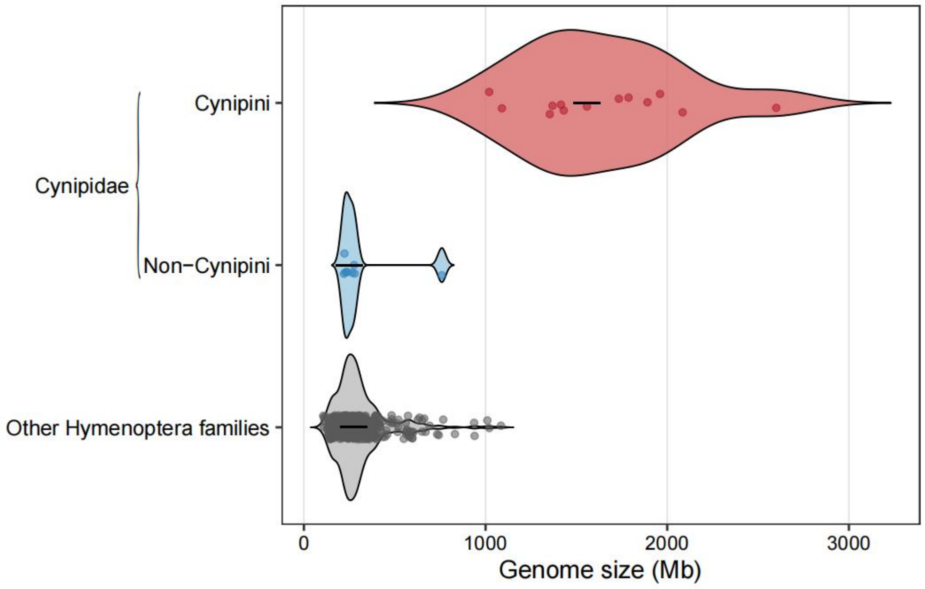

Supplementary Figure 1. Comparison of genome sizes among Cynipini, non-Cynipini, and other hymenopteran families.

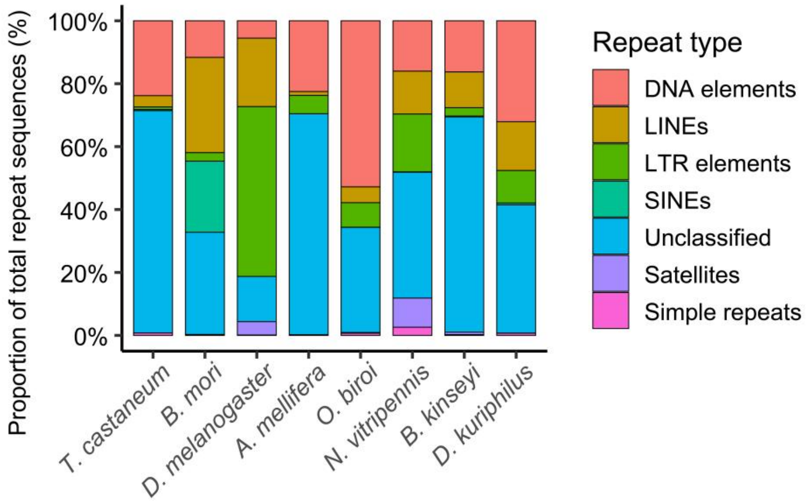

Supplementary Figure 2. Relative composition of repeat sequence types in the genomes of 8 insect species.

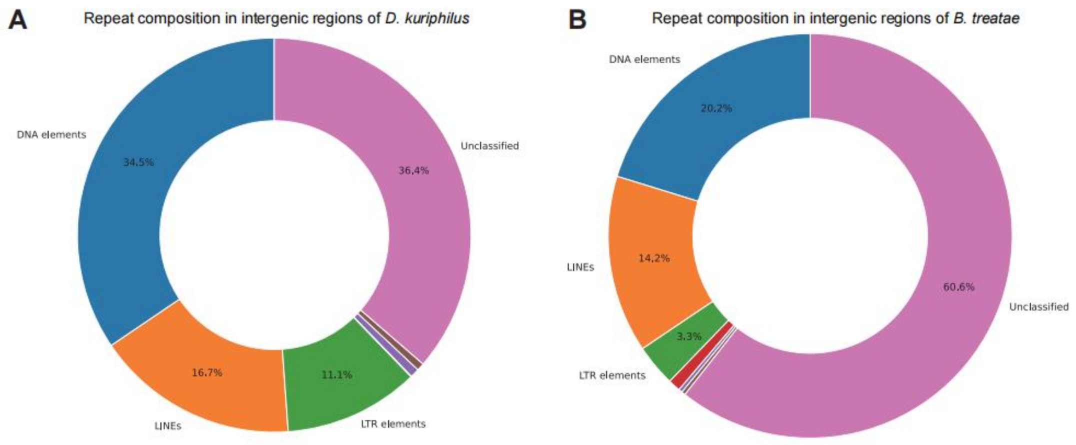

Supplementary Figure 3. Repeat composition in the intergenic regions of *Dryocosmus kuriphilus* and *Belonocnema kinseyi*.

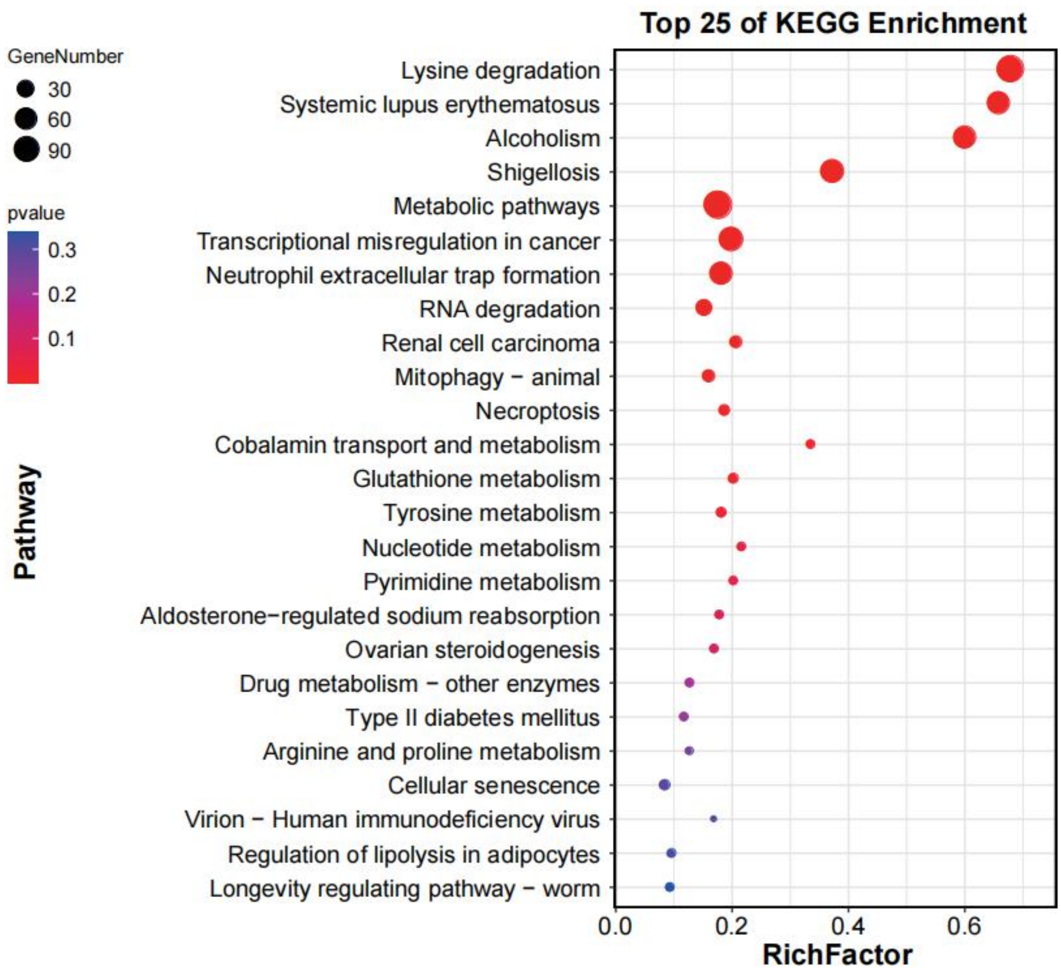

Supplementary Figure 4. KEGG pathway enrichment analysis of candidate genes.

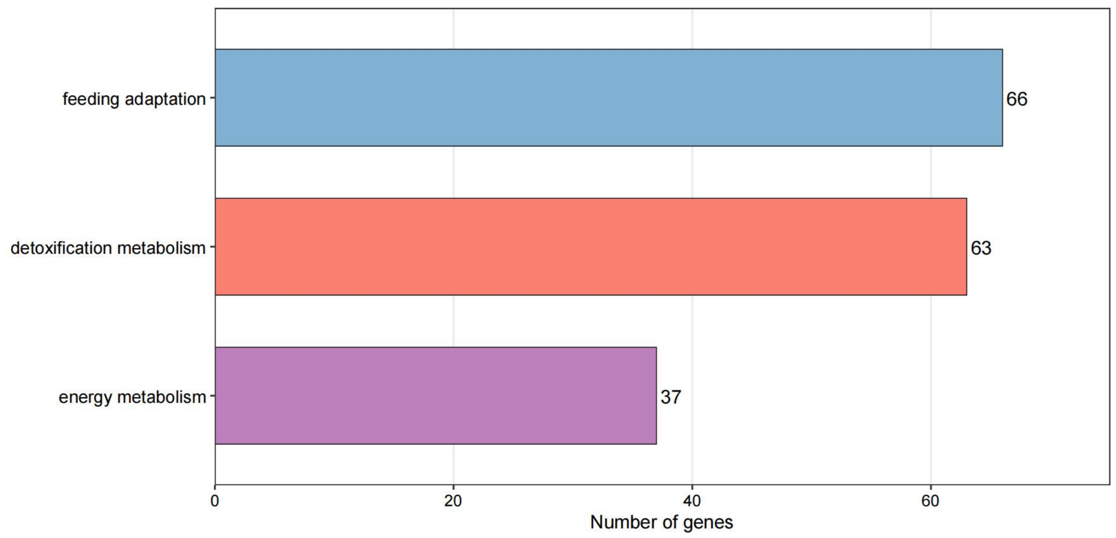

Supplementary Figure 5. Numbers of genes associated with feeding adaptation, detoxification metabolism, and energy metabolism.

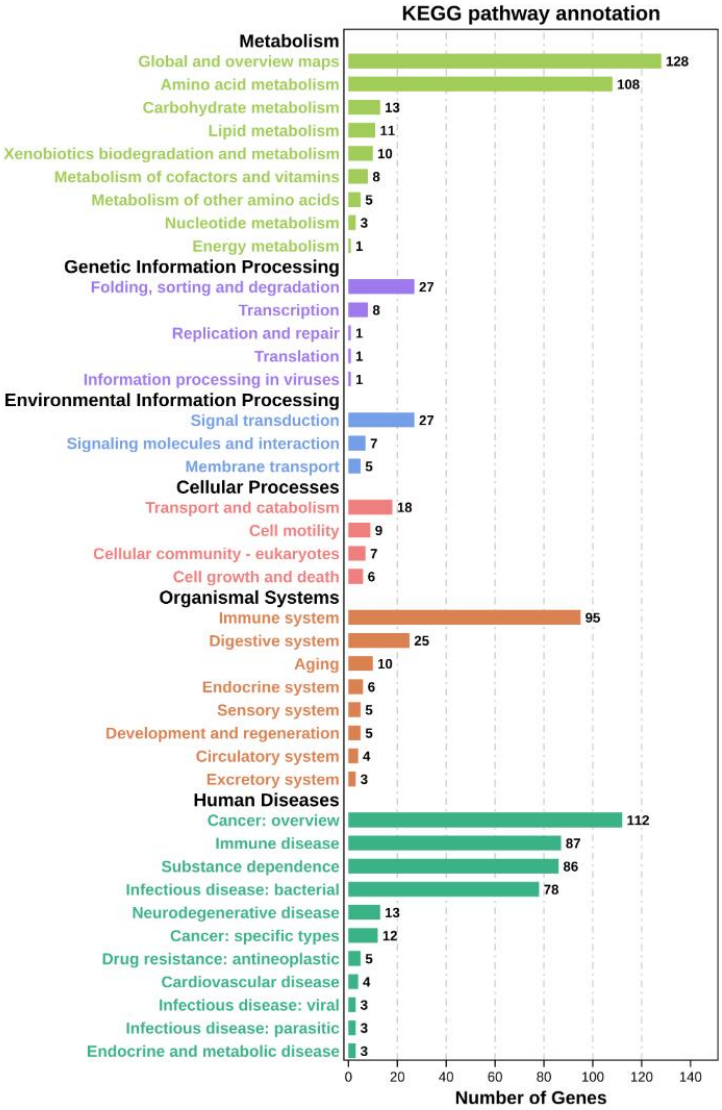

Supplementary Figure 6. KEGG pathway annotation and functional classification of candidate genes.

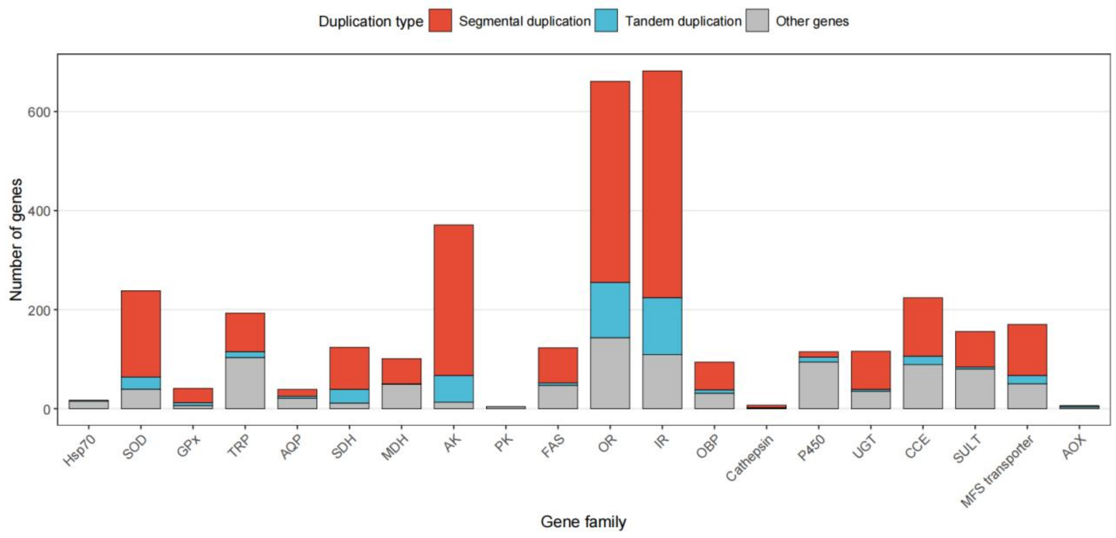

Supplementary Figure 7. Distribution of gene duplication types among different functional gene families.

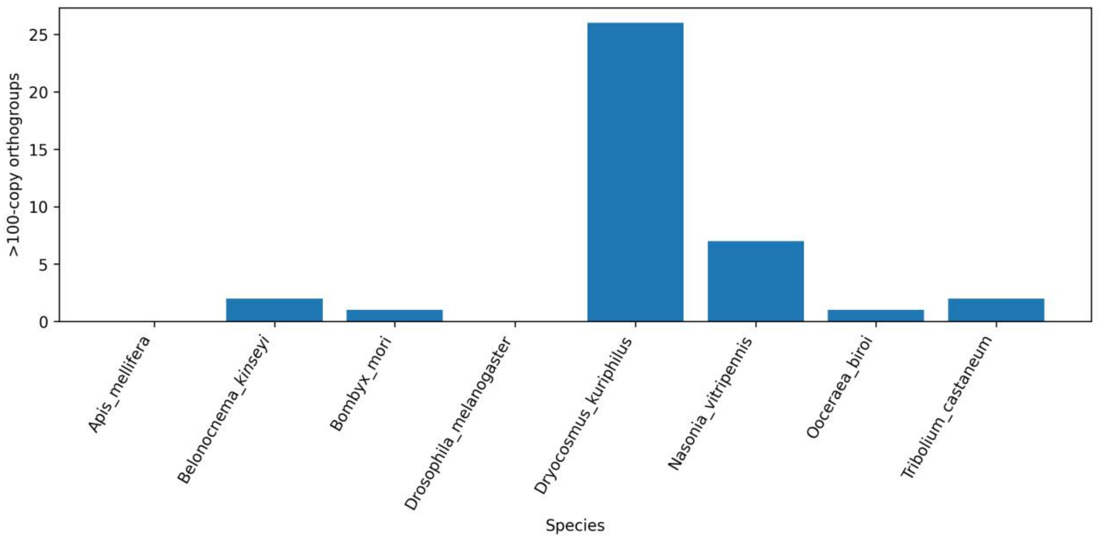

Supplementary Figure 8. Numbers of high-copy orthogroups (>100 within-species gene copies) across the eight insect species.

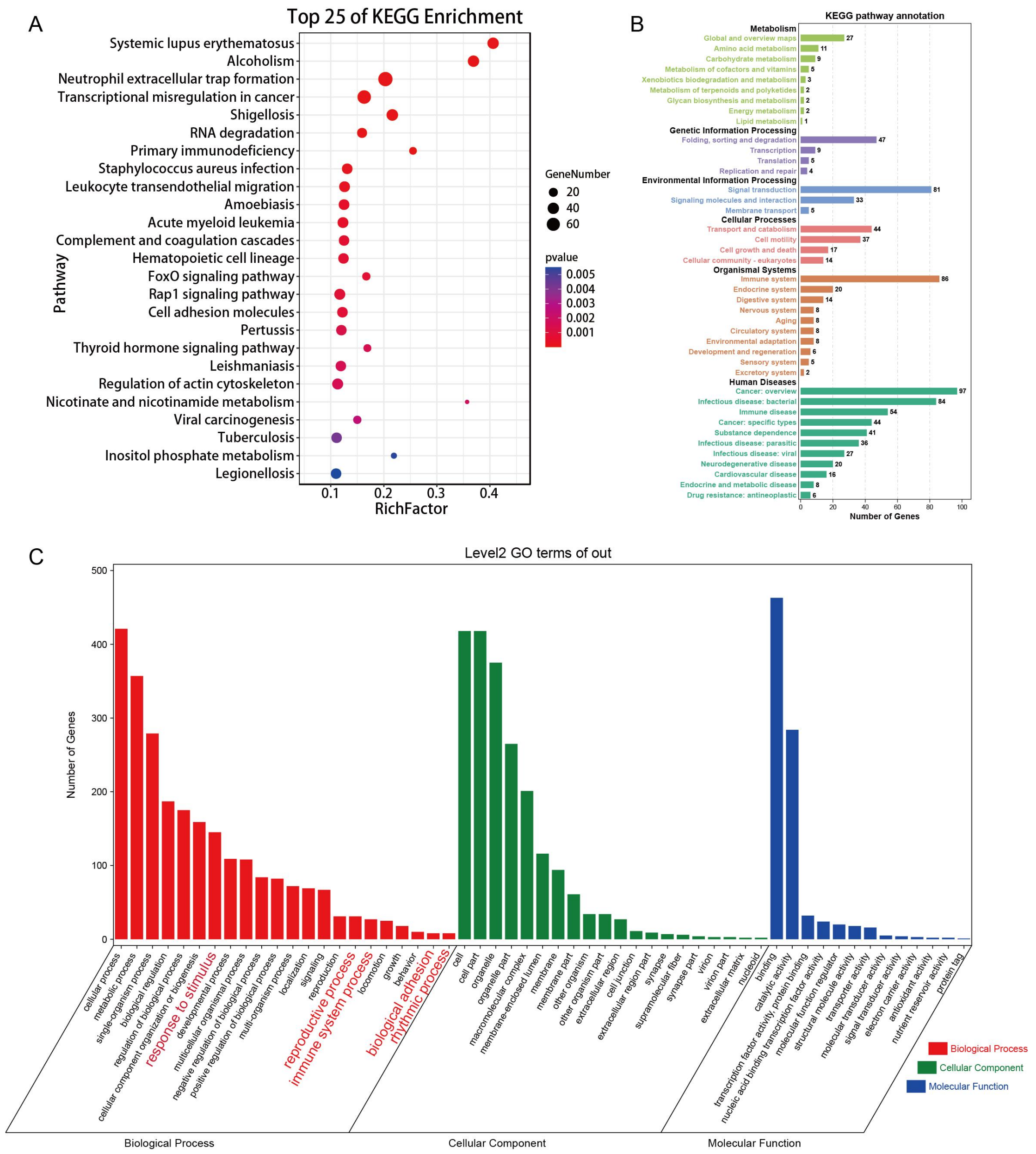

Supplementary Figure 9. Functional enrichment of genes associated with transposon-associated tandem duplication in the *D. kuriphilus* genome. A) KEGG enrichment of genes across level-3 metabolic pathways (P < 0.01). B) KEGG enrichment of genes across level-2 biological processes and functional categories (P < 0.01). C) GO enrichment analysis of the genes (P < 0.01).
